# *MTHFR* C677T TT genotype associated with poor blood pressure control in H-type hypertension without folic acid: a retrospective cohort study

**DOI:** 10.3389/fphar.2026.1885629

**Published:** 2026-09-04

**Authors:** Hong Cao, Jieru Wu, Yuan Yuan, Jing Li, Wenling Feng, Aliye Baierdi, Muyun Li, Yubo Wang, Jun Zhao

**Affiliations:** 1 State Key Laboratory of Pathogenesis, Prevention and Treatment of High Incidence Diseases in Central Asia, The First Affiliated Hospital of Xinjiang Medical University, Urumqi, China; 2 Xinjiang Key Laboratory of Clinical Drug Research, Urumqi, China; 3 Department of Pharmacy, Xinjiang Autonomous Region Hospital of Traditional Chinese Medicine, Urumqi, China; 4 Department of Pharmacy, Kashgar Prefecture Traditional Chinese Medicine Hospital, Kashgar, China; 5 School of Pharmacy, Xinjiang Medical University, Urumqi, China

**Keywords:** blood pressure control, folic acid, homocysteine, H-type hypertension, methylenetetrahydrofolate reductase, *MTHFR* C677T polymorphism, real-world data, retrospective cohort study

## Abstract

**Background:**

H-type hypertension, defined as primary hypertension combined with elevated plasma homocysteine (Hcy) ≥10 μmol/L, represents the predominant hypertensive phenotype in China. Although current guidelines recommend folic acid (FA) supplementation alongside antihypertensive therapy, a substantial treatment gap persists in routine clinical practice. The *MTHFR* C677T polymorphism is the principal genetic determinant of Hcy metabolism, yet its independent impact on blood pressure (BP) control in FA-untreated patients remains unknown.

**Methods:**

We conducted a single-centre, retrospective cohort study of 660 H-type hypertensive patients who received guideline-compliant antihypertensive therapy but no FA supplementation, enrolled from a tertiary hospital in Xinjiang, China (January 2019–December 2022). The primary outcome was BP control at follow-up (systolic BP < 140 mmHg and diastolic BP < 90 mmHg). Given the known dosage effect of *MTHFR* C677T on enzyme activity (CC > CT > TT), we evaluated three standard genetic models: a dominant model (CC vs. CT + TT), a recessive model (CC + CT vs. TT), and an additive model (CC vs. CT vs. TT). The recessive model was used as the primary contrast in PSM analysis to evaluate the high-risk TT homozygous group while maximising matched sample size.

**Results:**

The overall BP control rate was 61.5% (406/660). *MTHFR* genotype distribution was: CC 28.6%, CT 47.0%, TT 24.4%. In multivariable logistic regression using the recessive model, the TT genotype was associated with higher odds of uncontrolled BP compared with CC + CT carriers (OR = 1.89, 95% CI: 1.21–2.97; P = 0.005). LASSO-selected multivariable regression confirmed this association (OR = 1.83, 95% CI: 1.19–2.82; P = 0.006). After 1:2 PSM (CC + CT, n = 218; TT, n = 138), the TT genotype remained associated with higher odds of uncontrolled BP (OR = 1.75, 95% CI: 1.09–2.84; P = 0.022). Across all models, Grade 3 hypertension (OR range: 1.92–2.10 across the models), high psychological stress, high-salt dietary pattern, and physical inactivity were consistently identified as additional independent risk factors.

**Conclusion:**

In H-type hypertensive patients not receiving FA supplementation, the *MTHFR* 677 TT genotype was associated with poor BP control across multiple analytical models (adjusted OR range: 1.75–1.89 under recessive contrast; 2.06–2.48 under additive contrast). These findings suggest that the TT genotype may serve as a potential marker for identifying patients at higher risk of treatment resistance; whether genotype-guided FA supplementation improves BP control requires confirmation in prospective interventional studies.

## Introduction

Cardiovascular disease remains the leading cause of death worldwide, and hypertension is the single most prevalent and modifiable risk factor contributing to this burden (Roth et al., 2020). In China, an estimated 245 million adults have hypertension, and approximately 75% of these individuals have concomitant hyperhomocysteinaemia (elevated plasma Hcy ≥10 μmol/L), a combination termed “H-type hypertension” (GBD 2019 Risk Factors Collaborators, 2020; Wang et al., 2023). H-type hypertension is not simply an additive risk state; elevated Hcy and elevated blood pressure interact synergistically to impair endothelial function, promote oxidative stress, accelerate atherosclerosis, and amplify the risk of stroke, coronary artery disease, and cardiovascular mortality (Hypertension Group of the Chinese Society of Cardiology and A.C.G.o.t.C.S.o. Cardiology, 2017; Li et al., 2016; Chang et al., 2017). Large-scale prospective and epidemiological studies have confirmed that patients with H-type hypertension face a substantially greater risk of first stroke, coronary events, and all-cause death than individuals with hypertension alone, contributing a disproportionate burden of cardiovascular disease in China (Zhao et al., 2022; Zhang et al., 2021).

Given the central pathophysiological role of elevated Hcy in cardiovascular injury, the Chinese guidelines for the prevention and treatment of hypertension and associated expert consensus documents explicitly recommend that FA supplementation should be initiated alongside standard antihypertensive therapy in H-type hypertension (Li et al., 2016; Hu and Xu, 2008). FA is the essential cofactor for the remethylation of Hcy to methionine via the 5-methyltetrahydrofolate pathway; supplementation effectively reduces plasma Hcy concentrations. The landmark China Stroke Primary Prevention Trial (CSPPT), a multicentre randomised controlled trial enrolling 20,702 hypertensive adults, demonstrated that combined enalapril–FA therapy reduced the risk of first stroke by 21% compared with enalapril alone, establishing the evidence base for the “lower blood pressure and lower Hcy” strategy (Huo et al., 2015).

Despite this compelling evidence, a pronounced gap between guideline recommendations and clinical practice persists. The reasons for low FA prescription include limited physician awareness, lack of routine Hcy testing, and absence of genotype-guided therapy. Our preliminary audit of hospitalised patients in a regional tertiary centre revealed that the overwhelming majority of patients diagnosed with H-type hypertension were discharged without FA supplementation, receiving only conventional antihypertensive treatment. This widespread treatment gap raises a clinically important question: in the absence of FA, the key guideline-recommended protective intervention—what is the status of BP control in this large patient population, and which factors independently determine treatment outcomes?

A critical source of inter-individual variation in Hcy levels is the *MTHFR* C677T single-nucleotide polymorphism (rs1801133) (Qin et al., 2020). *MTHFR* (methylenetetrahydrofolate reductase) is the rate-limiting enzyme of the folate metabolic pathway, and the C-to-T transition at position 677 results in a thermolabile enzyme variant with approximately 70% lower activity in TT homozygotes compared with CC wild-type individuals (Yang et al., 2014). Consequently, TT carriers accumulate higher plasma Hcy concentrations than CT or CC carriers under equivalent dietary conditions. Accumulating evidence confirms that the *MTHFR* 677 TT genotype is a genetic susceptibility factor for essential hypertension and specifically for H-type hypertension (Niu et al., 2012; Pang et al., 2016). Furthermore, when patients receive FA therapy, the TT genotype significantly modifies the Hcy-lowering response, with TT carriers typically achieving greater absolute reductions in plasma Hcy (Qin et al., 2012; Huang et al., 2018). Importantly, patients with the *MTHFR* 677 TT genotype exhibit higher baseline BP and reduced BP response to standard antihypertensive regimens; notably, their BP is specifically amenable to riboflavin supplementation—the direct cofactor of *MTHFR*—suggesting a pathophysiological link between enzyme deficiency and vascular dysfunction (Wilson et al., 2013; Horigan et al., 2010).

However, the vast majority of studies examining the relationship between *MTHFR* C677T genotype and H-type hypertension outcomes have been conducted under the assumption that patients were receiving FA supplementation, comparing efficacy differences between genotype groups (Qin et al., 2012; Huang et al., 2018; Wilson et al., 2013). While several studies have examined genotype–FA interaction in treated cohorts, few have specifically focused on the real-world setting where FA supplementation is absent—a scenario that remains highly prevalent in routine clinical practice, particularly in resource-constrained regions. We hypothesise that the intrinsic metabolic dysfunction caused by the TT genotype, in the absence of exogenous FA correction—leads to persistent Hcy elevation that exacerbates vascular damage through endothelial dysfunction and oxidative stress, rendering BP harder to control with conventional antihypertensives and thereby directly worsening BP outcomes.

To address this knowledge gap, we designed a retrospective cohort study using real-world clinical data focused specifically on H-type hypertensive patients who were receiving guideline-compliant antihypertensive therapy but no FA supplementation. The objectives were: (1) to describe the BP control status and identify independent multifactorial determinants in this population; and (2) to rigorously test, using multiple complementary analytical approaches, whether the *MTHFR* 677 TT genotype is an independent risk factor for poor BP control in the absence of FA supplementation. These results are intended to provide the insight of genetic evidence for identifying higher-risk patients and to inform future studies of genotype-guided FA supplementation strategies in this specific patient subgroup.

## Methods

### Study design and ethical approval

This was a single-centre, retrospective cohort study. The study protocol was reviewed and approved by the Medical Ethics Committee of an affiliated hospital of Xinjiang Medical University (Approval Number: K202308-06). In accordance with the retrospective nature of the study and the minimal risk posed to participants, the Ethics Committee granted a waiver of individual patient informed consent. All patient data were fully anonymised prior to analysis to protect patient privacy. This report was prepared in accordance with the Strengthening the Reporting of Observational Studies in Epidemiology (STROBE) statement (Detailed STROBE checklist is reported in the Supplementary Material).

### Study setting and patient selection

Study participants were drawn from the Department of Cardiology at a large tertiary-level teaching hospital in the Xinjiang Uygur Autonomous Region, China. Using the hospital’s electronic medical record (EMR) system, we retrospectively and consecutively screened all hospitalised patients with H-type hypertension between 1 January 2019 and 31 December 2022.

The diagnosis of H-type hypertension required fulfilment of both criteria: (1) primary hypertension, diagnosed according to the 2018 Chinese Guidelines for the Prevention and Treatment of Hypertension (office systolic BP [SBP] ≥140 mmHg and/or diastolic BP [DBP] ≥90 mmHg on ≥3 separate occasions in the absence of antihypertensive medication, or a previously confirmed diagnosis with ongoing antihypertensive treatment); and (2) fasting plasma Hcy concentration ≥10 μmol/L.

Patients were included if they met all of the following criteria: (1) age ≥18 years; (2) *MTHFR* C677T genotyping completed during the index hospitalisation; (3) regular use of guideline-compliant antihypertensive medication for ≥1 month after discharge (drug class and dose per current guideline recommendations); and (4) availability of complete baseline clinical data and at least one valid BP measurement from clinic follow-up or structured telephone follow-up.

Patients were excluded if they: (1) used any formulation of FA supplementation or a fixed-dose combination antihypertensive product containing FA at any point during the hospitalisation or follow-up period; (2) had secondary hypertension, severe hepatic impairment (Child–Pugh Class C or above), severe chronic kidney disease (estimated glomerular filtration rate <30 mL/min/1.73 m^2^), active malignancy, thyroid dysfunction (hypo- or hyperthyroidism), or obstructive sleep apnoea syndrome; (3) were pregnant or breastfeeding; or (4) were lost to follow-up or had missing key follow-up data.

### Data collection

All data were obtained from two sources: the hospital EMR system and structured telephone follow-up interviews conducted by uniformly trained research staff using standardised questionnaires. FA non-supplementation was verified through EMR review, discharge medication review, and structured telephone follow-up questioning regarding FA-containing medications or supplements.

*MTHFR* C677T genotyping was performed on peripheral blood samples using a gene chip hybridisation method with the *MTHFR* (C677T) gene detection kit (PCR-chip hybridisation method; Shanghai BaiO Technology Co., Ltd., Shanghai, China). Hybridisation and signal detection were performed using the BR526-24 automatic hybridisation instrument and the BE-3.0 biochip reader (Shanghai BaiO Technology Co., Ltd., Shanghai, China). Positive and negative controls were included in each run, and the same method and platform were used throughout the study period.

Serum homocysteine concentration was measured on the AU5800 automated biochemical analyser using a commercial homocysteine assay kit manufactured by Meikang Biotechnology Co., Ltd (Ningbo, China). The assay was performed according to the manufacturer’s instructions. The coefficient of variation for repeat testing was required to be ≤ 5% at 10 ± 4 μmol/L and ≤3% at 20 ± 4 μmol/L. Serum Hcy was used as the available clinical measure of circulating Hcy.

Baseline data extracted from the EMR included: demographic information (age, sex, ethnicity); clinical characteristics (hypertension duration, hypertension grade [Grade 1, 2, or 3 per 2018 Chinese guidelines], family history of hypertension, height, weight, and comorbidities including coronary artery disease, atherosclerosis, cerebral infarction, type 2 diabetes mellitus [T2DM], and cardiac arrhythmia); laboratory results (fasting plasma Hcy concentration and *MTHFR* C677T genotype result obtained from clinical testing during hospitalisation); and in-hospital medications (antihypertensive drug classes: angiotensin-converting enzyme inhibitors [ACEIs], angiotensin II receptor blockers [ARBs], calcium channel blockers [CCBs], β-blockers, diuretics, α-blockers, and combination regimen details).

Follow-up data collected via structured telephone interview included: BP control status (the most recent SBP and DBP value recorded at a healthcare facility or a validated home BP measurement); lifestyle and behavioural factors including smoking status (never/passive/active), alcohol consumption history, self-reported psychological stress (frequent, occasional, or none), self-reported dietary salt habit (high-salt, moderate, or low-salt), and regular physical exercise frequency (defined as moderate-intensity exercise ≥30 min per session, categorised as: no exercise, <3 sessions/week, or ≥3 sessions/week); and confirmation of medication adherence. The median follow-up duration from discharge to BP outcome assessment was 18 months (IQR: 12–24 months).

### Variable definitions

The primary exposure variable was *MTHFR* C677T genotype, classified as CC (wild-type homozygote), CT (heterozygote), or TT (homozygous mutant). We evaluated three genetic models: the recessive model (TT vs. CC + CT), the dominant model (CT + TT vs. CC), and the additive model (CC vs. CT vs. TT as three separate groups). The recessive model was designated as the primary contrast and was used in PSM analysis. The dominant and additive models were secondary analyses. The primary outcome variable was BP control status, defined as a binary variable: controlled (SBP <140 mmHg and DBP <90 mmHg at follow-up, consistent with guideline thresholds for treated patients) or uncontrolled (failure to meet both criteria). Covariates included all potential confounders of BP control: age, sex, BMI, hypertension grade, baseline Hcy level (categorised as <15 or ≥15 μmol/L), antihypertensive combination regimen (monotherapy, dual therapy, or triple-and-above therapy), individual antihypertensive drug classes, and all lifestyle factors described above.

### Statistical analysis

Baseline characteristics are presented as median (interquartile range [IQR]) for non-normally distributed continuous variables, compared using the Mann–Whitney *U* test; and as mean ± standard deviation (SD) for normally distributed variables, compared by independent samples *t*-test. Categorical variables are expressed as frequency (percentage) and compared using the chi-squared (χ^2^) test or Fisher’s exact test as appropriate. Hardy-Weinberg equilibrium was assessed by comparing observed genotype frequencies against expected frequencies derived from allele frequencies under random mating assumptions, using a chi-squared goodness-of-fit test with 1 degree of freedom.

To identify and verify independent determinants of BP control, three analytical approaches were employed. First, a conventional multivariable binary logistic regression analysis was performed. All candidate variables with *P* < 0.10 in univariate analysis, together with clinically important variables regardless of univariate significance (age, baseline Hcy), were entered into the multivariable model. A backward stepwise selection procedure was applied. Collinearity was assessed using the variance inflation factor (VIF; threshold <10) and tolerance (threshold >0.1). Results are expressed as odds ratios (ORs) with 95% confidence intervals (CIs).

Second, to address potential multicollinearity and to objectively select the most parsimonious set of key predictors, least absolute shrinkage and selection operator (LASSO) penalised regression was applied using 10-fold cross-validation to determine the optimal regularisation parameter (λ.min = 0.01607). Ten-fold cross-validation was performed to select the optimal regularisation parameter. We chose λ.min (=0.01607; λ.1se = 0.04832) because our primary objective was variable selection for subsequent multivariable modelling rather than maximally penalised prediction, as λ.min minimises cross-validated prediction error and retains a richer feature set for clinical interpretation. The λ path and cross-validated AUC are provided in Supplementary Figure S2. Collinearity in the primary multivariable logistic regression was assessed using the variance inflation factor (VIF; all VIF <3.2; maximum tolerance >0.31), indicating no substantive multicollinearity among retained variables (Supplementary material).

Third, to more rigorously evaluate the independent effect of *MTHFR* C677T TT genotype and to control for potential selection bias arising from baseline group imbalances, propensity score matching (PSM) was performed. A logistic regression model was used to calculate the propensity score for each patient, with genotype grouping (CC + CT vs. TT) as the outcome and age, smoking status, alcohol consumption, BMI categories, hypertension grade, baseline Hcy level, psychological stress, dietary habits, and exercise frequency as matching covariates. Nearest-neighbour 1:2 matching was employed with a calliper width of 0.02. Covariate balance after matching was assessed using the standardised mean difference (SMD); SMD <0.10 was considered indicative of adequate balance. The association between TT genotype and BP control in the matched cohort was estimated by conditional logistic regression.

To formally decompose the total effect of the *MTHFR* TT genotype on BP control into an Hcy-mediated indirect effect and an Hcy-independent direct effect, causal mediation analysis was performed using the mediation framework. The exposure was TT genotype, the mediator was continuous plasma Hcy concentration (μmol/L), and the outcome was uncontrolled BP (binary). The mediator model was a linear regression of Hcy on TT genotype and covariates (age, sex, BMI category, hypertension grade, mental stress, dietary habit, and exercise frequency). The outcome model was a logistic regression of uncontrolled BP on TT genotype, Hcy, and the same covariate set. Average causal mediation effects (ACME), average direct effects (ADE), total effects, and the proportion mediated were estimated on the probability-difference scale with 95% confidence intervals derived from parametric bootstrap (2,000 replications). A *post hoc* power assessment was conducted for the primary TT versus CC + CT comparison and the PSM-matched cohort. The primary analysis had adequate power to detect the observed genotype effect, whereas subgroup analyses by individual ethnic group or antihypertensive drug class were considered exploratory because of limited sample size. All statistical analyses were performed using R software (version 4.4.3). All tests were two-sided, and *P* < 0.05 was considered statistically significant.

## Results

### Baseline characteristics

A total of 660 patients fulfilling all eligibility criteria were included. Overall, 406 patients (61.5%) achieved BP control at follow-up, while 254 (38.5%) had uncontrolled BP. The median age of the cohort was 52.0 years (IQR: 44.0–61.0), and 459 participants (69.5%) were male. *MTHFR* C677T genotype distribution was: CC 189 (28.6%), CT 310 (47.0%), and TT 161 (24.4%). Compared with the controlled group, patients in the uncontrolled group showed a significantly higher prevalence of TT genotype (29.1% vs. 21.4%, p = 0.041), Grade 3 hypertension (87.0% vs. 76.1%, *P* < 0.001), obesity (BMI ≥28 kg/m^2^, 42.1% vs. 33.7%, *P* = 0.045), frequent or occasional psychological stress (50.0% vs. 39.5%, p = 0.013), and high-salt dietary pattern (15.7% vs. 8.9%, p = 0.026). Regular exercise (>3 sessions/week, 28.3% vs. 36.7%, *P* = 0.032) and CCB use (84.3% vs. 75.6%, p = 0.011) were less frequent in the uncontrolled group. Triple-and-above antihypertensive combination therapy was also significantly more common among uncontrolled patients (44.9% vs. 35.5%, p = 0.002). The observed genotype distribution (CC: 189, CT: 310, TT: 161) did not deviate from Hardy–Weinberg equilibrium (Χ^2^ = 2.29, df = 1, p = 0.130), confirming the representativeness of the study sample. Detailed baseline characteristics are presented in Table 1.

**TABLE 1:** Baseline characteristics of the study population.

Variable	Category	Total (n = 660)	Control group (n = 406)	Uncontrolled group (n = 254)	W/χ^2^	P-value
Age, years	–	52.0 [44.0–61.0]	52.0 [44.0–61.8]	51.0 [44.0–59.0]	54537.5	0.212
BMI, kg/m^2^	–	26.8 [24.7–29.4]	26.3 [24.7–29.0]	27.2 [24.8–30.1]	46457	0.032*
Hcy, μmol/L	–	12.9 [11.5–14.9]	12.9 [11.5–14.6]	13.1 [11.3–15.0]	50534.5	0.667
Sex, n (%)	Male	459 (69.5%)	275 (67.7%)	184 (72.4%)	1.42	0.233
	Female	201 (30.5%)	131 (32.3%)	70 (27.6%)	​	​
Ethnicity, n (%)	Han	417 (63.2%)	264 (65.0%)	153 (60.2%)	3.06	0.217
	Uygur	192 (29.1%)	116 (28.6%)	76 (29.9%)	​	​
	Other	51 (7.7%)	26 (6.4%)	25 (9.8%)	​	​
Employment status, n (%)	Unemployed	280 (42.4%)	179 (44.1%)	101 (39.8%)	1.03	0.311
	Employed	380 (57.6%)	227 (55.9%)	153 (60.2%)	​	​
Education level, n (%)	Junior high school and below	145 (22.0%)	94 (23.2%)	51 (20.1%)	0.9	0.639
	Senior high school/Junior college	312 (47.3%)	188 (46.3%)	124 (48.8%)	​	​
	Bachelor’s degree and above	203 (30.8%)	124 (30.5%)	79 (31.1%)	​	​
Residence, n (%)	Rural	79 (12.0%)	46 (11.3%)	33 (13.0%)	0.27	0.605
	Urban	581 (88.0%)	360 (88.7%)	221 (87.0%)	​	​
Work type, n (%)	Retired unemployed	280 (42.4%)	179 (44.1%)	101 (39.8%)	3.73	0.292
	Manual worker	41 (6.2%)	24 (5.9%)	17 (6.7%)	​	​
	Mental worker	202 (30.6%)	114 (28.1%)	88 (34.6%)	​	​
	Mixed	137 (20.8%)	89 (21.9%)	48 (18.9%)	​	​
Duration of hypertension, n (%)	≤5 years	376 (57.0%)	233 (57.4%)	143 (56.3%)	0.36	0.948
	5–10 years	148 (22.4%)	91 (22.4%)	57 (22.4%)	​	​
	10–15 years	69 (10.5%)	43 (10.6%)	26 (10.2%)	​	​
	>15 years	67 (10.2%)	39 (9.6%)	28 (11.0%)	​	​
Hypertension grade, n (%)	Grade 1 and 2	130 (19.7%)	97 (23.9%)	33 (13.0%)	11.06	<0.001***
	Grade 3	530 (80.3%)	309 (76.1%)	221 (87.0%)	​	​
Risk classification, n (%)	Low/Moderate risk	22 (3.3%)	16 (3.9%)	6 (2.4%)	0.77	0.381
	High/Very high risk	638 (96.7%)	390 (96.1%)	248 (97.6%)	​	​
BMI classification, n (%)	<24	123 (18.6%)	74 (18.2%)	49 (19.3%)	6.21	0.045*
	24–28	293 (44.4%)	195 (48.0%)	98 (38.6%)	​	​
	≥28	244 (37.0%)	137 (33.7%)	107 (42.1%)	​	​
Hcy classification, n (%)	10–15	498 (75.5%)	314 (77.3%)	184 (72.4%)	1.77	0.184
	≥15	162 (24.5%)	92 (22.7%)	70 (27.6%)	​	​
Coronary heart disease, n (%)	No	572 (86.7%)	350 (86.2%)	222 (87.4%)	0.1	0.748
	Yes	88 (13.3%)	56 (13.8%)	32 (12.6%)	​	​
Atherosclerosis, n (%)	No	325 (49.2%)	198 (48.8%)	127 (50.0%)	0.05	0.82
	Yes	335 (50.8%)	208 (51.2%)	127 (50.0%)	​	​
Cerebral infarction, n (%)	No	430 (65.2%)	263 (64.8%)	167 (65.7%)	0.03	0.865
	Yes	230 (34.8%)	143 (35.2%)	87 (34.3%)	​	​
Coronary muscle bridge, n (%)	No	502 (76.1%)	307 (75.6%)	195 (76.8%)	0.06	0.807
	Yes	158 (23.9%)	99 (24.4%)	59 (23.2%)	​	​
Type 2 diabetes, n (%)	No	541 (82.0%)	341 (84.0%)	200 (78.7%)	2.57	0.109
	Yes	119 (18.0%)	65 (16.0%)	54 (21.3%)	​	​
Arrhythmia, n (%)	No	582 (88.2%)	359 (88.4%)	223 (87.8%)	0.01	0.905
	Yes	78 (11.8%)	47 (11.6%)	31 (12.2%)	​	​
Alcohol use history, n (%)	No	470 (71.2%)	292 (71.9%)	178 (70.1%)	0.18	0.674
	Yes	190 (28.8%)	114 (28.1%)	76 (29.9%)	​	​
Family history, n (%)	No	384 (58.2%)	241 (59.4%)	143 (56.3%)	0.48	0.487
	Yes	276 (41.8%)	165 (40.6%)	111 (43.7%)	​	​
Passive smoking, n (%)	No	356 (53.9%)	223 (54.9%)	133 (52.4%)	1.65	0.438
	Yes	80 (12.1%)	44 (10.8%)	36 (14.2%)	​	​
	Active smoking	224 (33.9%)	139 (34.2%)	85 (33.5%)	​	​
Mental stress, n (%)	Often	80 (12.1%)	40 (9.9%)	40 (15.7%)	8.68	0.013*
	Sometimes	207 (31.4%)	120 (29.6%)	87 (34.3%)	​	​
	No	373 (56.5%)	246 (60.6%)	127 (50.0%)	​	​
Dietary habit, n (%)	Salty	76 (11.5%)	36 (8.9%)	40 (15.7%)	7.26	0.026*
	Neutral	230 (34.8%)	146 (36.0%)	84 (33.1%)	​	​
	Light	354 (53.6%)	224 (55.2%)	130 (51.2%)	​	​
Vegetable/fruit intake, n (%)	Often	475 (72.0%)	302 (74.4%)	173 (68.1%)	3.08	0.215
	Sometimes	154 (23.3%)	87 (21.4%)	67 (26.4%)	​	​
	Rarely	31 (4.7%)	17 (4.2%)	14 (5.5%)	​	​
Exercise frequency, n (%)	No exercise	358 (54.2%)	204 (50.2%)	154 (60.6%)	6.89	0.032*
	≤3 times/week	81 (12.3%)	53 (13.1%)	28 (11.0%)	​	​
	>3 times/week	221 (33.5%)	149 (36.7%)	72 (28.3%)	​	​
Staying up late, n (%)	Often	71 (10.8%)	35 (8.6%)	36 (14.2%)	5.04	0.080
	Occasionally	159 (24.1%)	101 (24.9%)	58 (22.8%)	​	​
	No	430 (65.2%)	270 (66.5%)	160 (63.0%)	​	​
Sleep quality, n (%)	Good	326 (49.4%)	194 (47.8%)	132 (52.0%)	1.27	0.529
	Fair	239 (36.2%)	150 (36.9%)	89 (35.0%)	​	​
	Poor	95 (14.4%)	62 (15.3%)	33 (13.0%)	​	​
*MTHFR* genotype, n (%)	CC	189 (28.6%)	127 (31.3%)	62 (24.4%)	6.4	0.041*
	CT	310 (47.0%)	192 (47.3%)	118 (46.5%)	​	​
	TT	161 (24.4%)	87 (21.4%)	74 (29.1%)	​	​
*MTHFR* genotype (CC vs. CT + TT), n (%)	CT + TT	471 (71.4%)	279 (68.7%)	192 (75.6%)	3.28	0.070
	CC	189 (28.6%)	127 (31.3%)	62 (24.4%)	​	​
*MTHFR* genotype (CC + CT vs. TT), n (%)	CC + CT	499 (75.6%)	319 (78.6%)	180 (70.9%)	4.62	0.032*
	TT	161 (24.4%)	87 (21.4%)	74 (29.1%)	​	​
Arb, n (%)	No	109 (16.5%)	74 (18.2%)	35 (13.8%)	1.93	0.165
	Yes	551 (83.5%)	332 (81.8%)	219 (86.2%)	​	​
Acei, n (%)	No	644 (97.6%)	398 (98.0%)	246 (96.9%)	0.49	0.485
	Yes	16 (2.4%)	8 (2.0%)	8 (3.1%)	​	​
CCB, n (%)	No	139 (21.1%)	99 (24.4%)	40 (15.7%)	6.5	0.011*
	Yes	521 (78.9%)	307 (75.6%)	214 (84.3%)	​	​
Beta-blocker, n (%)	No	343 (52.0%)	212 (52.2%)	131 (51.6%)	0.01	0.936
	Yes	317 (48.0%)	194 (47.8%)	123 (48.4%)	​	​
Alpha-blocker, n (%)	No	608 (92.1%)	379 (93.3%)	229 (90.2%)	1.78	0.183
	Yes	52 (7.9%)	27 (6.7%)	25 (9.8%)	​	​
Diuretic, n (%)	No	614 (93.0%)	382 (94.1%)	232 (91.3%)	1.42	0.233
	Yes	46 (7.0%)	24 (5.9%)	22 (8.7%)	​	​
Lipid-lowering agents, n (%)	No	128 (19.4%)	78 (19.2%)	50 (19.7%)	0	0.961
	Yes	532 (80.6%)	328 (80.8%)	204 (80.3%)	​	​
Antithrombotic agents, n (%)	No	245 (37.1%)	152 (37.4%)	93 (36.6%)	0.02	0.896
	Yes	415 (62.9%)	254 (62.6%)	161 (63.4%)	​	​
Glucose-lowering agents, n (%)	No	549 (83.2%)	345 (85.0%)	204 (80.3%)	2.1	0.147
	Yes	111 (16.8%)	61 (15.0%)	50 (19.7%)	​	​
Medication combination, n (%)	Monotherapy	115 (17.4%)	86 (21.2%)	29 (11.4%)	12.1	0.002**
	Two drugs	287 (43.5%)	176 (43.3%)	111 (43.7%)	​	​
	Three or more drugs	258 (39.1%)	144 (35.5%)	114 (44.9%)	​	​
Final treatment duration, n (%)	≤1 year	96 (14.5%)	64 (15.8%)	32 (12.6%)	4.28	0.233
	1–3 years	466 (70.6%)	275 (67.7%)	191 (75.2%)	​	​
	3–5 years	62 (9.4%)	42 (10.3%)	20 (7.9%)	​	​
	>5 years	36 (5.5%)	25 (6.2%)	11 (4.3%)	​	​

Data are presented as mean ± SD, or n (%). Test statistic, W for continuous variables (Wilcoxon rank-sum test), Chi-square for categorical variables. Bold values indicate P < 0.05, considered statistically significant.

### Genetic model selection

Given the known dosage effect of *MTHFR* C677T on enzyme activity (CC > CT > TT), we evaluated three genetic models: additive (CC vs. CT vs. TT), recessive (CC + CT vs. TT), and dominant (CC vs. CT + TT). The additive model showed that, compared with CC homozygotes, the TT genotype was significantly associated with uncontrolled BP (OR = 2.48, 95% CI: 1.45–4.27; P = 0.001), whereas the CT genotype showed a directionally consistent but statistically non-significant trend (OR = 1.50, 95% CI: 0.97–2.31; P = 0.069) (Supplementary Table 4). The magnitude of association was greater for TT (OR = 2.48) than for CT (OR = 1.50), with CT not reaching statistical significance.

The recessive model (CC + CT vs. TT) was selected as the primary contrast for subsequent analyses and was also used in PSM analysis to maximize matched sample size. The dominant model (CC vs. CT + TT) was evaluated as a supplementary analysis (Supplementary Tables 3, 5, and 8) and showed consistent results, with the CC genotype conferring protection against uncontrolled BP (OR = 0.58, 95% CI: 0.38–0.88; P = 0.010).

### Multivariable logistic regression analysis

In univariate logistic regression using the recessive genetic model, the TT genotype (vs. CC + CT) was significantly associated with uncontrolled BP (OR = 1.51, 95% CI: 1.05–2.16; P = 0.025). Other variables significantly associated with uncontrolled BP in univariate analysis included Grade 3 hypertension (OR = 2.10, 95% CI: 1.38–3.27; P < 0.001), CCB use (OR = 1.73, 95% CI: 1.16–2.61; P = 0.009), triple-and-above antihypertensive combination regimen (OR = 2.35, 95% CI: 1.46–3.87; P < 0.001), frequent psychological stress, high-salt dietary pattern, and physical inactivity (>3 times/week vs. no exercise: OR = 0.64, 95% CI: 0.45–0.91; P = 0.013).

In multivariable logistic regression adjusted for demographic, clinical, pharmacological, and lifestyle factors (Table 2), the TT genotype remained independently associated with uncontrolled BP compared with CC + CT (adjusted OR = 1.89, 95% CI: 1.21–2.97; P = 0.005). Other independent predictors included Grade 3 hypertension (OR = 2.10, 95% CI: 1.27–3.58; P = 0.005), ethnic minority status (Uygur: OR = 1.65, 95% CI: 0.98–2.79, P = 0.063; Other: OR = 2.02, 95% CI: 1.03–3.97, P = 0.040 compared with Han ethnicity), absence of psychological stress (OR = 0.42, 95% CI: 0.22–0.78; P = 0.006 compared with frequent stress), and low-salt dietary pattern (neutral vs. salty: OR = 0.45, 95% CI: 0.25–0.81, P = 0.008; light vs. salty: OR = 0.53, 95% CI: 0.29–0.95, P = 0.032).

**TABLE 2:** Multivariable logistic regression analysis of uncontrolled blood pressure (recessive model: CC + CT vs. TT).

Variable	Category	Univariate logistic regression		Multivariate logistic regression	
		OR (95% CI)	P-value	OR (95% CI)	P-value
*MTHFR* genotype (CC + CT vs. TT)	CC + CT	​	​	​	​
	TT	1.51 (1.05–2.16)	0.025*	1.89 (1.21–2.97)	0.005**
Sex	Male	​	​	​	​
	Female	0.80 (0.56–1.13)	0.201	0.77 (0.48–1.26)	0.300
Ethnicity	Han	​	​	​	​
	Uygur	1.13 (0.79–1.60)	0.494	1.65 (0.98–2.79)	0.063
	Other	1.66 (0.92–2.98)	0.089	2.02 (1.03–3.97)	0.040*
Age, years	​	0.99 (0.98–1.01)	0.243	1.00 (0.98–1.03)	0.857
BMI, kg/m^2^	​	1.04 (0.99–1.08)	0.088	1.01 (0.96–1.06)	0.714
Employment status	Unemployed	​	​	​	​
	Employed	1.19 (0.87–1.64)	0.274	0.73 (0.40–1.31)	0.293
Education level	Junior high school and below	​	​	​	​
	Senior high school/Junior college	1.22 (0.81–1.84)	0.35	1.12 (0.69–1.82)	0.652
	Bachelor’s degree and above	1.17 (0.76–1.83)	0.477	1.03 (0.58–1.84)	0.921
Work type	Retired/Unemployed	​	​	​	​
	Manual worker	1.26 (0.64–2.43)	0.504	1.78 (0.77–4.10)	0.177
	Mental worker	1.37 (0.95–1.98)	0.097	1.25 (0.75–2.11)	0.390
	Mixed	0.96 (0.62–1.46)	0.836	NA	NA
Residence	Rural	​	​	​	​
	Urban	0.86 (0.53–1.39)	0.522	0.82 (0.48–1.43)	0.484
Duration of hypertension, years	≤5	​	​	​	​
	510	1.02 (0.69–1.51)	0.919	0.99 (0.63–1.54)	0.948
	1015	0.99 (0.57–1.66)	0.956	0.79 (0.42–1.46)	0.452
	>15	1.17 (0.68–1.98)	0.561	1.14 (0.58–2.22)	0.710
Hypertension grade	Grade 1 and 2	​	​	​	​
	Grade 3	2.10 (1.38–3.27)	<0.001***	2.10 (1.27–3.58)	0.005**
Risk classification	Low/Moderate risk	​	​	​	​
	High/Very high risk	1.70 (0.69–4.78)	0.277	0.97 (0.33–3.16)	0.958
Coronary heart disease	No	​	​	​	​
	Yes	0.90 (0.56–1.43)	0.661	0.88 (0.49–1.55)	0.656
Atherosclerosis	No	​	​	​	​
	Yes	0.95 (0.70–1.30)	0.758	1.13 (0.77–1.67)	0.537
Cerebral infarction	No	​	​	​	​
	Yes	0.96 (0.69–1.33)	0.799	1.08 (0.72–1.62)	0.704
Coronary muscle bridge	No	​	​	​	​
	Yes	0.94 (0.65–1.35)	0.735	0.85 (0.56–1.29)	0.454
Type 2 diabetes mellitus (T2DM)	No	​	​	​	​
	Yes	1.42 (0.95–2.11)	0.089	0.99 (0.34–2.88)	0.980
Arrhythmia	No	​	​	​	​
	Yes	1.06 (0.65–1.71)	0.808	0.93 (0.53–1.60)	0.787
Alcohol use history	No	​	​	​	​
	Yes	1.09 (0.77–1.54)	0.611	1.03 (0.64–1.66)	0.896
Family history	No	​	​	​	​
	Yes	1.13 (0.83–1.56)	0.438	1.10 (0.76–1.59)	0.631
Final treatment duration, years	≤1	​	​	​	​
	13	1.39 (0.88–2.23)	0.164	1.72 (1.03–2.92)	0.039*
	35	0.95 (0.48–1.87)	0.888	1.24 (0.58–2.65)	0.576
	>5	0.88 (0.37–1.98)	0.762	1.08 (0.42–2.68)	0.877
ARB	Not used	​	​	​	​
	Used	1.39 (0.91–2.18)	0.136	0.98 (0.34–2.86)	0.975
ACEI	Not used	​	​	​	​
	Used	1.62 (0.59–4.45)	0.342	1.72 (0.37–8.06)	0.489
CCB	Not used	​	​	​	​
	Used	1.73 (1.16–2.61)	0.009**	1.18 (0.43–3.24)	0.749
Beta-blocker	Not used	​	​	​	​
	Used	1.03 (0.75–1.40)	0.872	0.74 (0.30–1.84)	0.519
Alpha-blocker	Not used	​	​	​	​
	Used	1.53 (0.86–2.71)	0.141	1.01 (0.42–2.43)	0.974
Diuretic	Not used	​	​	​	​
	Used	1.51 (0.82–2.76)	0.179	1.07 (0.46–2.47)	0.867
Combination regimen	Monotherapy	​	​	​	​
	Two drugs	1.87 (1.16–3.07)	0.011*	1.97 (0.69–5.74)	0.208
	Three or more drugs	2.35 (1.46–3.87)	<0.001***	2.49 (0.37–16.97)	0.348
Lipid-lowering agents	Not used	​	​	​	​
	Used	0.97 (0.65–1.45)	0.881	0.91 (0.55–1.51)	0.700
Glucose-lowering agents	Not used	​	​	​	​
	Used	1.39 (0.92–2.09)	0.12	1.34 (0.44–4.07)	0.599
Passive smoking	No	​	​	​	​
	Yes	1.37 (0.84–2.24)	0.206	1.42 (0.79–2.53)	0.240
	Active smoking	1.03 (0.73–1.45)	0.887	0.77 (0.47–1.25)	0.287
Mental stress	Often	​	​	​	​
	Sometimes	0.73 (0.43–1.22)	0.224	0.69 (0.39–1.24)	0.220
	No	0.52 (0.32–0.84)	0.008**	0.42 (0.22–0.78)	0.006**
Dietary habit	Salty	​	​	​	​
	Neutral	0.52 (0.31–0.87)	0.014*	0.45 (0.25–0.81)	0.008**
	Light	0.52 (0.32–0.86)	0.011*	0.53 (0.29–0.95)	0.032*
Vegetable/fruit intake	Often	​	​	​	​
	Sometimes	1.34 (0.93–1.94)	0.116	1.24 (0.81–1.89)	0.311
	Rarely	1.44 (0.68–2.99)	0.331	1.32 (0.56–3.05)	0.517
Exercise frequency (times/week)	No exercise	​	​	​	​
	≤3	0.70 (0.42–1.15)	0.165	0.68 (0.37–1.22)	0.203
	>3	0.64 (0.45–0.91)	0.013*	0.71 (0.46–1.09)	0.119
Staying up late	Often	​	​	​	​
	Occasionally	0.56 (0.32–0.98)	0.044	0.52 (0.27–0.99)	0.047
	No	0.58 (0.35–0.96)	0.032	0.59 (0.31–1.11)	0.103
Sleep quality	Good	​	​	​	​
	Fair	0.87 (0.62–1.23)	0.434	0.86 (0.58–1.26)	0.428
	Poor	0.78 (0.48–1.25)	0.313	0.55 (0.31–0.95)	0.035
Hcy, μmol/L	​	1.01 (0.99–1.02)	0.507	0.99 (0.97–1.01)	0.221

Bold values indicate P < 0.05, considered statistically significant.

### LASSO-penalized regression analysis

To address potential multicollinearity and to objectively identify the most parsimonious set of key predictors, least absolute shrinkage and selection operator (LASSO) penalized regression was applied as a sensitivity analysis. Ten-fold cross-validation was performed to determine the optimal regularization parameter. We selected λ.min = 0.01607 (λ.1se = 0.04832) to minimize cross-validated prediction error while retaining a clinically interpretable feature set. Collinearity diagnostics confirmed no substantial multicollinearity among retained variables in the primary multivariable model (all variance inflation factors [VIF] <3.2; minimum tolerance >0.31).

LASSO retained 15 predictors from the original 27 candidate variables, including *MTHFR* genotype (CC + CT vs. TT), ethnicity, BMI, hypertension grade, T2DM, CCB use, beta-blocker use, antihypertensive combination regimen, glucose-lowering drug use, psychological stress, dietary pattern, vegetable/fruit intake, exercise frequency, and sleep quality.

In multivariable logistic regression using only LASSO-selected predictors (Table 3), under the recessive model, the TT genotype remained significantly associated with uncontrolled BP (adjusted OR = 1.83, 95% CI: 1.19–2.82; P = 0.006 compared with CC + CT). The LASSO-adjusted OR was highly consistent with the primary multivariable regression estimate (OR = 1.89, Table 2), confirming the robustness of the observed genotype–BP control association.

**TABLE 3:** Univariate and multivariable logistic regression analysis of LASSO-selected predictors associated with uncontrolled blood pressure (CC + CT vs. TT).

Variable	Category	Univariate logistic regression		Multivariate logistic regression	
		OR (95% CI)	P-value	OR (95% CI)	P-value
*MTHFR* genotype (CC + CT vs. TT)	CC + CT	​	​	​	​
	TT	1.51 (1.05–2.16)	0.025*	1.83 (1.19–2.82)	0.006**
Ethnicity	Han	​	​	​	​
	Uygur	1.13 (0.79–1.60)	0.494	1.38 (0.88–2.17)	0.166
	Other	1.66 (0.92–2.98)	0.089	1.87 (0.98–3.55)	0.055
BMI, kg/m^2^	​	1.04 (0.99–1.08)	0.088	1.01 (0.97–1.06)	0.548
Hypertension grade	Grade 1 and 2	​	​	​	​
	Grade 3	2.10 (1.38–3.27)	<0.001***	1.92 (1.22–3.09)	0.006**
T2DM	No	​	​	​	​
	Yes	1.42 (0.95–2.11)	0.089	1.01 (0.36–2.82)	0.99
CCB	Not used	​	​	​	​
	Used	1.73 (1.16–2.61)	0.009**	1.22 (0.69–2.17)	0.497
β-blocker	Not used	​	​	​	​
	Used	1.03 (0.75–1.40)	0.872	0.72 (0.44–1.17)	0.186
Antihypertensive combination	Monotherapy	​	​	​	​
	Two drugs	1.87 (1.16–3.07)	0.011*	1.83 (1.03–3.31)	0.043*
	Three or more drugs	2.35 (1.46–3.87)	<0.001***	2.37 (1.06–5.40)	0.037*
Glucose-lowering drugs	Not used	​	​	​	​
	Used	1.39 (0.92–2.09)	0.12	1.27 (0.44–3.68)	0.662
Mental stress	Often	​	​	​	​
	Sometimes	0.73 (0.43–1.22)	0.224	0.68 (0.39–1.19)	0.176
	None	0.52 (0.32–0.84)	0.008**	0.45 (0.26–0.78)	0.005**
Dietary pattern	Salty	​	​	​	​
	Neutral	0.52 (0.31–0.87)	0.014*	0.47 (0.26–0.83)	0.009**
	Light	0.52 (0.32–0.86)	0.011*	0.54 (0.31–0.93)	0.027*
Vegetable/fruit intake	Often	​	​	​	​
	Sometimes	1.34 (0.93–1.94)	0.116	1.17 (0.78–1.74)	0.448
	Rarely	1.44 (0.68–2.99)	0.331	1.38 (0.62–3.03)	0.419
Exercise frequency, times/week	No exercise	​	​	​	​
	≤3	0.70 (0.42–1.15)	0.165	0.69 (0.39–1.21)	0.197
	>3	0.64 (0.45–0.91)	0.013*	0.68 (0.45–1.02)	0.063
Sleep quality	Good	​	​	​	​
	Fair	0.87 (0.62–1.23)	0.434	0.85 (0.59–1.23)	0.39
	Poor	0.78 (0.48–1.25)	0.313	0.58 (0.34–0.98)	0.046*
Hcy, μmol/L	​	1.01 (0.99–1.02)	0.507	0.99 (0.97–1.01)	0.205

Bold values indicate P < 0.05, considered statistically significant.

In the additive model using LASSO-selected predictors (Supplementary Table 6), the TT genotype showed a strong association (OR = 2.06, 95% CI: 1.28–3.34; P = 0.003 compared with CC), whereas the CT genotype showed a borderline significant association (OR = 1.43, 95% CI: 0.95–2.17; P = 0.089), further supporting TT homozygosity as the primary driver of the observed effect. Alternative genetic models under LASSO adjustment yielded consistent findings (Supplementary Table 7).

### Propensity score matching analysis

To obtain a more robust estimate of the independent effect of *MTHFR* TT genotype on BP control while minimizing confounding from baseline group differences, 1:2 propensity score matching (PSM) was performed using the recessive genetic model (CC + CT vs. TT). A logistic regression model was used to calculate the propensity score for each patient, with TT genotype status as the outcome and age, smoking status, alcohol consumption, BMI categories, hypertension grade, baseline Hcy level (< 15 vs. ≥15 μmol/L), psychological stress, dietary habits, and exercise frequency as matching covariates. Nearest-neighbor 1:2 matching was employed with a caliper width of 0.02.

After 1:2 matching, the matched cohort comprised 356 patients (CC + CT: n = 218; TT: n = 138). Post-matching covariate balance was substantially improved, with standardized mean differences (SMD) < 0.10 for most variables (Supplementary Table 2). However, plasma Hcy classification (10–15 vs. ≥15 μmol/L) remained statistically imbalanced between matched groups (CC + CT: 33.5% with Hcy ≥15 μmol/L vs. TT: 47.8%; P = 0.010; SMD = 0.294). Hcy classification was retained as a covariate in the subsequent conditional logistic regression model. Twenty-nine patients (8.1%) were excluded from conditional logistic regression due to non-informative matched strata. The final analytical sample comprised 327 patients (CC + CT: n = 189; TT: n = 138).

In the PSM-matched cohort, conditional logistic regression (Table 4) demonstrated that TT genotype remained a significant and independent predictor of uncontrolled BP compared with the CC + CT reference group (OR = 1.75, 95% CI: 1.09–2.84; P = 0.022), after adjusting for residual Hcy imbalance and other covariates. Grade 3 hypertension (OR = 1.92, 95% CI: 1.05–3.59; P = 0.037) and dietary pattern remained independently significant: compared with a high-salt diet, neutral dietary pattern was associated with 61% lower odds of uncontrolled BP (OR = 0.39, 95% CI: 0.17–0.84; P = 0.017), and light dietary pattern was associated with 66% lower odds (OR = 0.34, 95% CI: 0.16–0.71; P = 0.005). Triple-and-above antihypertensive regimen was associated with nearly four-fold higher odds of uncontrolled BP in the matched cohort (OR = 3.99, 95% CI: 1.28–13.27; P = 0.020).

**TABLE 4:** Conditional logistic regression analysis of uncontrolled blood pressure with MTHFR genotype grouping (CC + CT vs. TT) in the propensity score-matched cohort.

Variable	Category	Univariate logistic regression		Multivariate logistic regression	
		OR (95% CI)	P-value	OR (95% CI)	P-value
*MTHFR* genotype (CC + CT vs. TT)	CC + CT	​	​	​	​
	TT	1.57 (1.06–2.33)	0.024*	1.75 (1.09–2.84)	0.022*
Ethnicity	Han	​	​	​	​
	Uygur	1.39 (0.88–2.19)	0.156	1.16 (0.59–2.26)	0.664
	Other	1.80 (0.95–3.42)	0.072	2.37 (1.05–5.51)	0.040*
BMI classification	<24	​	​	​	​
	24–28	0.71 (0.44–1.13)	0.145	0.69 (0.37–1.28)	0.241
	≥28	0.94 (0.58–1.53)	0.794	1.16 (0.62–2.17)	0.651
Hypertension grade	Grade 1 and 2	​	​	​	​
	Grade 3	1.98 (1.26–3.19)	0.004**	1.92 (1.05–3.59)	0.037*
T2DM	Yes	​	​	​	​
	No	0.99 (0.35–2.77)	0.983	0.92 (0.17–4.84)	0.923
CCB	Not used	​	​	​	​
	Used	1.21 (0.69–2.16)	0.506	1.13 (0.49–2.60)	0.768
Beta-blocker	Used	​	​	​	​
	Not used	0.71 (0.43–1.16)	0.169	0.63 (0.31–1.24)	0.183
Antihypertensive combination regimen	Monotherapy	​	​	​	​
	Two drugs	1.78 (1.00–3.22)	0.052	1.78 (0.79–4.18)	0.175
	Three or more drugs	2.34 (1.05–5.31)	0.040*	3.99 (1.28–13.27)	0.020*
Glucose-lowering drugs	Used	​	​	​	​
	Not used	1.28 (0.44–3.72)	0.651	1.04 (0.19–5.86)	0.961
Psychological stress	Often	​	​	​	​
	Sometimes	0.67 (0.38–1.16)	0.15	0.73 (0.35–1.51)	0.393
	Rarely	0.44 (0.25–0.77)	0.004**	0.57 (0.27–1.17)	0.126
Dietary pattern	Salty	​	​	​	​
	Neutral	0.49 (0.27–0.86)	0.013*	0.39 (0.17–0.84)	0.017*
	Light	0.55 (0.31–0.95)	0.032*	0.34 (0.16–0.71)	0.005**
Vegetable and fruit intake	Often	​	​	​	​
	Sometimes	1.16 (0.77–1.73)	0.477	0.86 (0.47–1.55)	0.609
	Rarely	1.31 (0.59–2.87)	0.499	0.96 (0.34–2.65)	0.936
Exercise frequency (times/week)	No exercise	​	​	​	​
	<3 times/week	0.73 (0.41–1.26)	0.262	0.94 (0.42–2.07)	0.879
	>3 times/week	0.71 (0.47–1.07)	0.1	0.65 (0.38–1.13)	0.126
Sleep quality	Good	​	​	​	​
	Fair	0.85 (0.59–1.23)	0.392	0.64 (0.38–1.07)	0.09
	Poor	0.59 (0.34–0.99)	0.048*	0.57 (0.27–1.15)	0.123

Bold values indicate P < 0.05, considered statistically significant.

### Causal mediation analysis

The TT genotype was strongly associated with elevated Hcy in the mediator model (adjusted β = 9.59 μmol/L, P < 0.001). In the fully adjusted outcome model including both TT genotype and Hcy, Hcy was not a significant predictor of uncontrolled BP (OR = 0.99, 95% CI: 0.97–1.01; P = 0.276), whereas TT remained significant (OR = 1.73, 95% CI: 1.14–2.63; P = 0.011). Mediation decomposition confirmed that the indirect effect through Hcy did not reach significance (ACME = −0.023, 95% CI: −0.071 to 0.021; P = 0.288), while the direct effect independent of Hcy was significant (ADE = 0.124, 95% CI: 0.029–0.218; P = 0.009), yielding a total effect of 0.101 (95% CI: 0.017–0.189; P = 0.020). The proportion of the total effect mediated by Hcy was −22.2% (P = 0.300), confirming that plasma Hcy did not account for the observed TT-BP association. These results support the characterisation of the *MTHFR* 677 TT genotype as an independent risk factor, operating through Hcy-independent pathways (Table 5; Supplementary Tables 11, 12).

**TABLE 5:** Causal mediation analysis of the *MTHFR* TT Genotype–BP control association.

Effect	Estimate	95% CI	P
Total effect	0.101	0.017–0.189	0.020
ADE (direct, Hcy-independent)	0.124	0.029–0.218	0.009
ACME (indirect, via Hcy)	−0.023	−0.071–0.021	0.288
Proportion mediated	−0.222	−1.681–0.293	0.300

Estimates are on the probability-difference (risk difference) scale. ADE, average direct effect; ACME, average causal mediation effect. analyses adjusted for age, sex, BMI category, hypertension grade, mental stress, dietary habit, and exercise frequency. Parametric bootstrap with 2,000 replications.

The negative proportion mediated (−22.2%) suggests a potential suppression effect, wherein the TT genotype and Hcy may operate through partially opposing pathways on BP control, or that unmeasured confounders related to both TT and Hcy obscure the mediation pathway. This finding underscores the complexity of the genotype-Hcy-BP relationship and supports the existence of Hcy-independent mechanisms.

### Additional sensitivity analysis: inverse probability weighting

As an additional sensitivity analysis using a different causal inference approach, inverse probability weighting (IPW) was applied under the additive genetic model to assess the robustness of findings across all three genotypes. IPW-weighted multivariable logistic regression yielded effect estimates consistent with unweighted and PSM-adjusted analyses: compared with CC, the TT genotype was associated with more than two-fold higher odds of uncontrolled BP (OR = 2.09, 95% CI: 1.62–2.70; P < 0.001), while the CT genotype showed a smaller but statistically significant association (OR = 1.43, 95% CI: 1.11–1.83; P = 0.005) (Supplementary Table 9). The consistency of findings across IPW, PSM, LASSO, and unweighted regression strengthens confidence in the robustness of the observed *MTHFR* C677T–BP control association (Supplementary Table 9).

## Discussion

This study provides real-world evidence suggesting that the *MTHFR* C677T genotype may be associated with BP control among H-type hypertensive patients who were not receiving FA supplementation. Using the recessive genetic model (TT vs. CC + CT) as the primary contrast, the 677 TT genotype was consistently associated with higher odds of uncontrolled BP across three complementary analytical approaches: multivariable logistic regression (OR = 1.89, 95% CI: 1.21–2.97; P = 0.005), LASSO-penalized regression (OR = 1.83, 95% CI: 1.19–2.82; P = 0.006), and propensity score matching-adjusted conditional logistic regression (OR = 1.75, 95% CI: 1.09–2.84; P = 0.022). The adjusted odds ratios were highly consistent across models (range: 1.75–1.89), with a maximum relative difference of less than 8%.

To comprehensively evaluate the genetic architecture of this association, we examined three genetic models: additive (CC vs. CT vs. TT), recessive (CC + CT vs. TT), and dominant (CC vs. CT + TT). *MTHFR* enzymatic activity decreases in a genotype-dependent manner, with approximately 65% of normal activity retained in CT heterozygotes and 30% in TT homozygotes (Frosst et al., 1995). The additive model showed that, compared with CC, the TT genotype was strongly associated with uncontrolled BP (OR = 2.48, 95% CI: 1.45–4.27; P = 0.001), whereas the CT genotype showed a weaker, non-significant trend (OR = 1.50, 95% CI: 0.97–2.31; P = 0.069) (Supplementary Table 4). This pattern suggests the observed effect is predominantly driven by TT homozygosity.

Inverse probability weighting confirmed these findings under the additive model (TT vs. CC: OR = 2.09, 95% CI: 1.62–2.70, P < 0.001; CT vs. CC: OR = 1.43, 95% CI: 1.11–1.83, P = 0.005) (Supplementary Table 9). The recessive model yielded consistent results (OR = 1.89, 95% CI: 1.21–2.97; P = 0.005), whereas the dominant model showed a protective effect for the CC genotype (OR = 0.58, 95% CI: 0.38–0.88; P = 0.010) (Supplementary Table 5). These findings are consistent with meta-analytic evidence showing stronger associations under the recessive model (TT vs. CC + CT: OR = 1.51, 95% CI: 1.31–1.74) than the dominant model (CT + TT vs. CC: OR = 1.37, 95% CI: 1.24–1.52) (Meng et al., 2021; Supplementary Table 10), and extend this evidence to the context of BP control in FA-untreated H-type hypertension.

Although these findings remained directionally consistent after adjustment for clinical, pharmacological, and lifestyle covariates, they should be interpreted as associative rather than causal because of the retrospective design and the use of routine clinical laboratory and BP-monitoring data. The causal mediation analysis suggested that the observed TT–BP association was not mediated by measured plasma Hcy levels (ACME = −0.023, P = 0.288), but rather operated through Hcy-independent pathways (ADE = 0.124, P = 0.009). However, residual confounding and unmeasured folate-related factors cannot be excluded. These findings suggest that *MTHFR* C677T genotype may contribute to risk stratification in FA-untreated H-type hypertension, while prospective studies are needed to confirm whether genotype-guided interventions improve BP outcomes.

This interpretation is consistent with previous evidence linking the 677 TT genotype to higher blood pressure, increased hypertension risk, and poorer blood pressure control during antihypertensive treatment (Ward et al., 2020). A genotype-targeted trial further reported a blood pressure-lowering response to riboflavin, an essential *MTHFR* cofactor, among hypertensive individuals with the TT genotype (Wilson et al., 2012). Nevertheless, the most recent Cochrane review concluded that evidence for the antihypertensive effect of riboflavin remains very uncertain (Bradbury et al., 2025). Therefore, the dominant model may be useful for broader genetic risk assessment, whereas the recessive model may better identify TT homozygotes who are particularly susceptible to poor blood pressure control. Although these findings support further investigation of genotype-informed nutritional strategies, current evidence is insufficient to recommend folic acid or riboflavin supplementation for blood pressure control solely on the basis of *MTHFR* genotype.

The mechanistic rationale for our principal finding is well-grounded. The TT variant of *MTHFR* C677T results in a thermolabile enzyme with markedly reduced catalytic activity, impeding the conversion of 5,10-methylenetetrahydrofolate to 5-methyltetrahydrofolate—the primary methyl donor for the remethylation of Hcy to methionine (Qin et al., 2020). In the absence of exogenous FA to supplement this metabolic bottleneck, Hcy accumulates chronically in TT carriers at levels substantially above those in CC or CT individuals. Sustained hyperhomocysteinaemia mediates vascular injury through multiple interrelated pathways: impaired endothelial nitric oxide bioavailability, augmented superoxide generation, enhanced NADPH oxidase activity, and structural arterial remodelling resulting in increased vascular stiffness (Ganguly and Alam, 2015; Tyagi et al., 2005). These mechanisms elevate BP and may attenuate the responsiveness of the vasculature to conventional antihypertensive agents. In support of this biological hypothesis, prior clinical evidence has demonstrated that hypertensive patients with the *MTHFR* 677 TT genotype appear more resistant to routine antihypertensive drug therapy, and that their elevated BP is specifically responsive to riboflavin supplementation—the direct cofactor of *MTHFR*—independently of conventional antihypertensive agents (Wilson et al., 2013; Horigan et al., 2010). Our real-world observational data extend this concept to the H-type hypertension context and suggests that the inherent metabolic vulnerability associated with the TT genotype may contribute to inferior BP control outcomes in the context of absent FA therapy, though mediation through elevated Hcy cannot be formally excluded based on observational data alone.

Our findings are biologically consistent with, but clinically distinct from, prior meta-analytic evidence. A meta-analysis by Niu et al. (Niu et al., 2012) reported that the *MTHFR* 677 TT genotype is strongly associated with an increased risk of developing hypertension and hypertension in pregnancy in Chinese populations (OR > 1.3). Yang et al. (Yang et al., 2014) similarly confirmed this association in a meta-analysis of over 10,000 subjects. However, these studies addressed disease susceptibility (i.e., the probability of developing hypertension), whereas our study addresses treatment response (i.e., the probability of failing to control BP despite treatment). This distinction is clinically important because it directly informs therapeutic decision-making for already-diagnosed patients.

Beyond the genetic finding, our results quantify the independent contributions of modifiable lifestyle factors to BP control in H-type hypertensives. Frequent psychological stress was associated with an approximately 90% increase in the odds of uncontrolled BP, consistent with well-established neuroendocrine mechanisms linking chronic stress to sympathetic over-activation, renin–angiotensin–aldosterone system upregulation, and sustained BP elevation (Kivimäki and Steptoe, 2018). A high-salt dietary pattern independently doubled the risk of uncontrolled BP relative to a low-salt diet, in agreement with meta-analyses and international guideline recommendations for sodium restriction in hypertension management (World Health Organization, 2012). Regular exercise was associated with approximately 35% lower odds of uncontrolled BP, aligning with evidence that physical activity reduces BP through both haemodynamic and neurohumoral mechanisms (Paluch et al., 2022). An important contextual consideration is that hyperhomocysteinaemia and adverse lifestyle exposures may interact synergistically through shared pathways of oxidative stress and endothelial injury (Xiao et al., 2023). This suggests that in H-type hypertensive patients, the threshold for lifestyle intervention should be even lower than in non-H-type patients, and that optimisation of diet, physical activity, and stress management constitutes an integral—not supplementary—component of comprehensive management.

The LASSO model achieved a cross-validated AUC of 0.669, indicating modest discrimination. This level of performance may be useful for exploratory risk stratification but is insufficient as a stand-alone clinical prediction tool. Incorporating direct folate and vitamin B12 measurements, ambulatory BP monitoring, and objective adherence data may improve future predictive models. The estimate for triple-and-above therapy in the PSM model should be interpreted cautiously because of its wide confidence interval and likely instability in the matched cohort. Ethnicity was retained as an important covariate because *MTHFR* allele frequencies and BP-control determinants may differ across ethnic groups in Xinjiang. Any ethnic association should be interpreted cautiously, as it may reflect a mixture of genetic background, socioeconomic context, healthcare access, dietary patterns, and residual confounding rather than an independent biological effect alone.

The high rate of uncontrolled BP (38.5%) in our cohort reflects both the intrinsic clinical challenge of H-type hypertension and the consequences of the prevailing treatment gap. National survey data have documented improvements in hypertension awareness, treatment, and control rates in China over recent decades, but overall BP control remains suboptimal, with control rates in the range of 15%–30% in general population studies and somewhat higher in treated hospital-based cohorts (Zhang et al., 2023). Our rate of 61.5% control among treated hospitalised patients, while higher than community estimates, nonetheless indicates that more than one-third of patients on active therapy fail to achieve target BP—a finding with profound public health implications. The root cause is partly structural: guideline-recommended FA therapy for H-type hypertension is seldom initiated, representing a failure at the level of physician awareness, prescription practice, and healthcare system implementation (Cabana et al., 1999). Our data provide quantitative evidence to motivate action at all of these levels.

From a precision medicine perspective, our findings provide a genetic rationale for risk-stratified management of H-type hypertension. The CSPPT enrolled approximately 20,702 hypertensive patients and demonstrated that FA supplementation reduced first stroke risk by 21%.11 Given that approximately 24.4% of our cohort carried the TT genotype—a proportion consistent with reported prevalences in Chinese populations—TT carriers likely represent the subgroup with the greatest absolute benefit from combined antihypertensive and FA therapy. Our PSM-adjusted OR of 1.75 for uncontrolled BP directly and quantitatively supports prospective evaluation of genotype-guided folic acid intervention in TT carriers. This is consistent with the growing pharmacogenomics literature advocating for the use of genetic information to individualise cardiovascular treatment strategies (Lugtenberg et al., 2009; Johnson and Cavallari, 2013). Although routine *MTHFR* genotyping for hypertension management remains impractical in many primary care settings and faces challenges related to evidence standardisation (Arnett and Claas, 2009), the H-type hypertension phenotype, characterised by a clearly elevated Hcy threshold and a readily accessible genetic test—represents one of the most tractable opportunities for implementing pharmacogenomic guidance in routine cardiovascular care in China. Future pharmacogenetic studies should examine whether *MTHFR* C677T genotype modifies BP response to specific antihypertensive drug classes and whether genotype-guided FA or riboflavin supplementation improves BP control in H-type hypertension.

Several biological and methodological limitations should be acknowledged. This study examined only the *MTHFR* C677T polymorphism and did not assess other variants involved in folate and homocysteine metabolism, such as *MTRR* A66G and *MTR* A2756G; thus, a broader polygenic approach may better capture the genetic contribution to folate metabolism, Hcy regulation, and blood pressure control (Wang et al., 2017). In the propensity score analysis, the TT group was considerably smaller than the combined CC + CT group, limiting the number of suitable matches and the ability to achieve complete covariate balance. Despite these limitations, the consistent findings across additional covariate adjustment and sensitivity analyses lend support to the observed association. Mediation analysis did not identify a significant indirect effect through measured serum Hcy, but downstream Hcy-related or other folate-pathway mechanisms cannot be excluded. Future studies with larger and more balanced genotype groups, broader genetic profiling, and repeated measurements of folate-related biomarkers are warranted to further clarify the underlying mechanisms.

As a single-centre retrospective study based on EMR-derived routine clinical data, the findings may not be generalisable to all geographic regions or healthcare settings, and residual unmeasured confounding cannot be excluded. Although *MTHFR* C677T genotyping and serum Hcy measurement were performed using established laboratory methods with routine quality-control procedures, the retrospective design limited the availability of complete laboratory quality-assurance information, such as reagent lot records and between-batch variability, which could not be systematically evaluated. BP values were derived from clinic measurements or home BP values documented during follow-up, with some home measurements being patient-reported, introducing potential heterogeneity in BP assessment. Because the measurement source could not be reliably distinguished for all participants, a sensitivity analysis restricted to clinic-based BP measurements could not be performed. The absence of 24-h ambulatory BP monitoring also limited the assessment of white-coat and masked hypertension, and outcome misclassification cannot be completely excluded. In addition, baseline folate and vitamin B12 concentrations were unavailable, and the sample size limited subgroup analyses by ethnicity and antihypertensive drug class. Nevertheless, the observed association between the TT genotype and uncontrolled BP was directionally consistent across multiple complementary analytical approaches. Prospective multicentre studies incorporating standardised genotyping, biomarker assessment, and BP monitoring protocols are needed to validate these findings.

## Conclusion

This real-world retrospective cohort study suggests that, among H-type hypertensive patients not receiving FA supplementation, the *MTHFR* C677T TT genotype is associated with poorer BP control across complementary analytical models. The dominant and recessive genetic-model analyses suggest that the strongest signal is observed under the recessive TT model, whereas CC may be relatively protective. However, given the retrospective design, reliance on routine clinical laboratory data, mixed BP measurement sources, and the absence of baseline folate and vitamin B12 measurements, these findings should be interpreted as hypothesis-generating rather than definitive evidence of causality. *MTHFR* genotyping may help identify patients who warrant closer BP monitoring and further evaluation, but whether genotype-guided FA, riboflavin, or antihypertensive strategies improve BP control requires confirmation in prospective interventional studies.

## Data Availability

Due to the inclusion of patient personal privacy and confidential information, the raw data are not publicly available. The processed data are retained by the research investigators. Researchers wishing to access the relevant data should contact the corresponding authors.
