# Supplementary material for: MTHFR C677T TT genotype associated with poor blood pressure control in H-type hypertension without folic acid: a retrospective cohort study
Source: Front Pharmacol. 2026 Sep 4;17:1885629. doi: 10.3389/fphar.2026.1885629 (PMC13585575; doi:10.3389/fphar.2026.1885629)
Supplement: Supplementary file 1 [file Supplementaryfile1.docx]

# Table 1 STROBE Checklist: Observational Cohort Study

| **Item No.** | **STROBE Recommendation** | **Reported in Manuscript** | **Location / Comment** |
| --- | --- | --- | --- |
| 1 | Indicate the study’s design (e.g., cohort, case-control, cross-sectional) | Yes | Abstract, Methods |
| 2 | Describe the setting, locations, and relevant dates | Yes | Methods: "tertiary hospital in Xinjiang... Jan 2019Dec 2022" |
| 3 | Describe eligibility criteria for participants | Yes | Methods: inclusion/exclusion criteria listed |
| 4 | Explain the recruitment process and sources of participants | Yes | Methods: "retrospectively screened from EMR" |
| 5 | Describe methods of follow-up | Yes | Methods: "structured telephone interview" |
| 6 | Describe methods of assessment of exposure and outcomes | Yes | Methods: MTHFR genotyping, BP measurement, Hcy lab method |
| 7 | Describe how confounding variables were assessed | Yes | Methods: list of covariates; PSM variables specified |
| 8 | Describe methods of statistical analysis | Yes | Methods: LASSO λ.min = 0.01607, λ.1se = 0.04832; VIF <3.2, tolerance >0.31 |
| 9 | Describe any methods used to address potential sources of bias | Yes | PSM, LASSO, multivariable regression |
| 10 | Describe the flow of participants through the study | Partial | Total n=660, but no CONSORT-style flow diagram; PSM matching loss (356→327) explained in text |
| 11 | Give numbers of individuals at each stage of the study | Yes | Results: 660 included; 406 controlled/254 uncontrolled; PSM: 218+138 |
| 12 | Give characteristics of study participants (e.g., demographics, clinical features) | Yes | Table 1 |
| 13 | Report numbers of outcome events or summary measures | Yes | BP control rate 61.5% (406/660) |
| 14 | Report measures of association (e.g., OR, RR) with 95% CIs | Yes | Tables 25 |
| 15 | Report adjusted estimates and their precision | Yes | All multivariable/PSM models |
| 16 | Report results of any sensitivity analyses | Partial | LASSO and PSM serve as sensitivity analyses, but no formal sensitivity test (e.g., excluding T2DM) |
| 17 | Report any subgroup analyses | Partial | Genotype subgroups (CC vs. TT; CC+CT vs. TT) reported, but no formal interaction test (e.g., genotype × stress) |
| 18 | Report limitations of the study | Yes | Discussion: 4 limitations listed |
| 19 | Discuss the generalisability (external validity) of the findings | Yes | Discussion: single-center, Xinjiang population |
| 20 | Discuss the interpretation of findings considering the strengths and limitations | Yes | Discussion: triple-validation, real-world gap |
| 21 | Discuss the implications for clinical practice, policy, or future research | Yes | Discussion: precision medicine, FA prioritization |
| 22 | Give the source of funding and the role of the funders | Yes | Funding section |

Table 2 Baseline characteristics before and after Propensity Score Matching

TT vs (CC + CT)

| **Variable** | **Before Matching** | | | | **After Matching** | | | |
| --- | --- | --- | --- | --- | --- | --- | --- | --- |
|  | **CC+CT**  **(n=499)** | **TT**  **(n=161)** | **P** | **SMD** | **CC+CT**  **(n=218)** | **TT**  **(n=138)** | **P** | **SMD** |
| Age, years | 53.0 (44.0-61.0) | 51.0 (44.0-60.0) | 0.342 | -0.097 | 52.0 ± 12.5 | 51.8 ± 10.7 | 0.861 | -0.031 |
| Passive smoking, |  |  | 0.085 |  |  |  | 0.573 |  |
| No | 280 (56.1%) | 76 (47.2%) |  | -0.089 | 110 (50.5%) | 63 (45.7%) |  | -0.018 |
| Yes | 61 (12.2%) | 19 (11.8%) |  | -0.004 | 30 (13.8%) | 18 (13.0%) |  | -0.011 |
| Active | 158 (31.7%) | 66 (41.0%) |  | 0.093 | 78 (35.8%) | 57 (41.3%) |  | 0.029 |
| Alcohol use history | 144 (28.9%) | 46 (28.6%) | 1.000 | -0.003 | 57 (26.1%) | 38 (27.5%) | 0.868 | 0.000 |
| BMI |  |  | 0.062 |  |  |  | 0.735 |  |
| <24 kg/m² | 83 (16.6%) | 40 (24.8%) |  | 0.082 | 54 (24.8%) | 34 (24.6%) |  | 0.000 |
| 24~28 kg/m² | 225 (45.1%) | 68 (42.2%) |  | -0.029 | 85 (39.0%) | 59 (42.8%) |  | 0.040 |
| ≥28 kg/m² | 191 (38.3%) | 53 (32.9%) |  | -0.054 | 79 (36.2%) | 45 (32.6%) |  | -0.040 |
| Hypertension grade |  |  | 0.187 |  |  |  | 0.775 |  |
| 1 or 2 | 92 (18.4%) | 38 (23.6%) |  | -0.052 | 48 (22.0%) | 33 (23.9%) |  | -0.018 |
| 3 | 407 (81.6%) | 123 (76.4%) |  |  | 170 (78.0%) | 105 (76.1%) |  |  |
| Hcy classification |  |  | **<0.001***** |  |  |  | **0.010*** |  |
| 10-15μmol/L | 426 (85.4%) | 72 (44.7%) |  | 0.407 | 145 (66.5%) | 72 (52.2%) |  | 0.294 |
| ≥15μmol/L | 73 (14.6%) | 89 (55.3%) |  |  | 73 (33.5%) | 66 (47.8%) |  |  |
| Mental stress |  |  | 0.246 |  |  |  | 0.638 |  |
| Often | 55 (11.0%) | 25 (15.5%) |  | 0.045 | 29 (13.3%) | 23 (16.7%) |  | 0.022 |
| Sometimes | 155 (31.1%) | 52 (32.3%) |  | 0.012 | 73 (33.5%) | 42 (30.4%) |  | -0.040 |
| No | 289 (57.9%) | 84 (52.2%) |  | -0.057 | 116 (53.2%) | 73 (52.9%) |  | 0.018 |
| Exercise frequency |  |  | **0.010*** |  |  |  | 0.651 |  |
| No exercise | 287 (57.5%) | 71 (44.1%) |  | -0.134 | 93 (42.7%) | 65 (47.1%) |  | 0.040 |
| ≤3 times/week | 59 (11.8%) | 22 (13.7%) |  | 0.018 | 29 (13.3%) | 15 (10.9%) |  | -0.025 |
| >3 times/week | 153 (30.7%) | 68 (42.2%) |  | 0.116 | 96 (44.0%) | 58 (42.0%) |  | -0.015 |
| Dietary habit |  |  | 0.166 |  |  |  | 0.697 |  |
| Salty | 51 (10.2%) | 25 (15.5%) |  | 0.053 | 30 (13.8%) | 15 (10.9%) |  | -0.033 |
| Neutral | 174 (34.9%) | 56 (34.8%) |  | -0.001 | 81 (37.2%) | 51 (37.0%) |  | 0.004 |
| Light | 274 (54.9%) | 80 (49.7%) |  | -0.052 | 107 (49.1%) | 72 (52.2%) |  | 0.029 |

Table 3 Baseline characteristics before and after Propensity Score Matching

CC vs (CT + TT)

| **Variable** | **Before Matching** | | | | **After Matching** | | | |
| --- | --- | --- | --- | --- | --- | --- | --- | --- |
|  | TT+CT  (n=471) | CC  (n=189) | P | SMD | TT+CT  (n=303) | CC  (n=188) | P | SMD |
| Age, years | 51.6 ± 11.5 | 54.5 ± 12.1 | **0.004**** | 0.241 | 53.1 ± 11.3 | 54.4 ± 12.1 | 0.233 | 0.026 |
| Passive smoking, |  |  | 0.126 |  |  |  | 0.801 |  |
| No | 245 (52.0%) | 111 (58.7%) |  | 0.067 | 182 (60.1%) | 110 (58.5%) |  | -0.013 |
| Yes | 64 (13.6%) | 16 (8.5%) |  | -0.051 | 29 (9.6%) | 16 (8.5%) |  | -0.003 |
| Active | 162 (34.4%) | 62 (32.8%) |  | -0.016 | 92 (30.4%) | 62 (33.0%) |  | 0.016 |
| Alcohol use history | 142 (30.1%) | 48 (25.4%) | 0.261 | -0.048 | 80 (26.4%) | 48 (25.5%) | 0.914 | -0.005 |
| BMI |  |  | 0.264 |  |  |  | 0.720 |  |
| <24 kg/m² | 91 (19.3%) | 32 (16.9%) |  | -0.024 | 57 (18.8%) | 32 (17.0%) |  | -0.005 |
| 24~28 kg/m² | 215 (45.6%) | 78 (41.3%) |  | -0.044 | 131 (43.2%) | 78 (41.5%) |  | 0.000 |
| ≥28 kg/m² | 165 (35.0%) | 79 (41.8%) |  | 0.068 | 115 (38.0%) | 78 (41.5%) |  | 0.005 |
| Hypertension grade |  |  | 0.555 |  |  |  | 0.793 |  |
| 1 or 2 | 96 (20.4%) | 34 (18.0%) |  | -0.024 | 59 (19.5%) | 34 (18.1%) |  | 0.011 |
| 3 | 375 (79.6%) | 155 (82.0%) |  |  | 244 (80.5%) | 154 (81.9%) |  |  |
| Hcy classification |  |  | **<0.001***** |  |  |  | 0.701 |  |
| 10-15μmol/L | 329 (69.9%) | 169 (89.4%) |  | -0.196 | 266 (87.8%) | 168 (89.4%) |  | 0.008 |
| ≥15μmol/L | 142 (30.1%) | 20 (10.6%) |  |  | 37 (12.2%) | 20 (10.6%) |  |  |
| Mental stress |  |  | **0.011*** |  |  |  | 0.639 |  |
| Often | 63 (13.4%) | 17 (9.0%) |  | -0.044 | 33 (10.9%) | 17 (9.0%) |  | -0.013 |
| Sometimes | 159 (33.8%) | 48 (25.4%) |  | -0.084 | 84 (27.7%) | 48 (25.5%) |  | 0.008 |
| No | 249 (52.9%) | 124 (65.6%) |  | 0.127 | 186 (61.4%) | 123 (65.4%) |  | 0.005 |
| Exercise frequency |  |  | **0.002**** |  |  |  | 0.202 |  |
| No exercise | 235 (49.9%) | 123 (65.1%) |  | 0.152 | 172 (56.8%) | 122 (64.9%) |  | 0.016 |
| ≤3 times/week | 64 (13.6%) | 17 (9.0%) |  | -0.046 | 33 (10.9%) | 17 (9.0%) |  | 0.000 |
| >3 times/week | 172 (36.5%) | 49 (25.9%) |  | -0.106 | 98 (32.3%) | 49 (26.1%) |  | -0.016 |
| Dietary habit |  |  | 0.972 |  |  |  | 0.835 |  |
| Salty | 54 (11.5%) | 22 (11.6%) |  | 0.002 | 31 (10.2%) | 22 (11.7%) |  | 0.005 |
| Neutral | 163 (34.6%) | 67 (35.4%) |  | 0.008 | 104 (34.3%) | 66 (35.1%) |  | 0.003 |
| Light | 254 (53.9%) | 100 (52.9%) |  | -0.010 | 168 (55.4%) | 100 (53.2%) |  | -0.008 |

Table 4. Multivariable Logistic Regression Analysis of Uncontrolled Blood Pressure with Alternative MTHFR Genotype Grouping (CC vs. CT vs. TT)

| **Variable** | **Category** | **Univariate logistic regression** | | **Multivariate logistic regression** | |
| --- | --- | --- | --- | --- | --- |
|  |  | **OR (95% CI)** | P-value | **OR (95% CI)** | **P-value** |
| MTHFR genotype | CC |  |  |  |  |
|  | CT | 1.26 (0.86-1.85) | 0.236 | 1.50 (0.97-2.31) | 0.069 |
|  | TT | 1.74 (1.13-2.70) | **0.012*** | 2.48 (1.45-4.26) | **0.001***** |
| Sex | Male |  |  |  |  |
|  | Female | 0.80 (0.56-1.13) | 0.201 | 0.77 (0.47-1.25) | 0.298 |
| Ethnicity | Han |  |  |  |  |
|  | Uygur | 1.13 (0.79-1.60) | 0.494 | 1.73 (1.02-2.95) | **0.042*** |
|  | Other | 1.66 (0.92-2.98) | 0.089 | 2.23 (1.13-4.44) | **0.021*** |
| Age, years | – | 0.99 (0.98-1.01) | 0.243 |  |  |
| BMI, kg/m² | – | 1.04 (0.99-1.08) | 0.088 | 0.71 (0.39-1.28) | 0.256 |
| Employment status | Unemployed |  |  |  |  |
|  | Employed | 1.19 (0.87-1.64) | 0.274 | 1.10 (0.68-1.79) | 0.700 |
| Education level | Junior high school and below |  |  | 1.01 (0.57-1.80) | 0.981 |
|  | Senior high school / Junior college | 1.22 (0.81-1.84) | 0.350 |  |  |
|  | Bachelor's degree and above | 1.17 (0.76-1.83) | 0.477 | 1.75 (0.75-4.04) | 0.188 |
| Work type | Retired / Unemployed |  |  | 1.32 (0.78-2.23) | 0.297 |
|  | Manual worker | 1.26 (0.64-2.43) | 0.504 | NA | NA |
|  | Mental worker | 1.37 (0.95-1.98) | 0.097 |  |  |
|  | Mixed | 0.96 (0.62-1.46) | 0.836 | 0.81 (0.47-1.41) | 0.459 |
| Residence | Rural |  |  | 1.00 (0.98-1.03) | 0.839 |
|  | Urban | 0.86 (0.53-1.39) | 0.522 | 1.01 (0.96-1.06) | 0.746 |
| Duration of hypertension, years | ≤5 |  |  |  |  |
|  | 5–10 | 1.02 (0.69-1.51) | 0.919 | 0.98 (0.62-1.54) | 0.940 |
|  | 10–15 | 0.99 (0.57-1.66) | 0.956 | 0.81 (0.43-1.50) | 0.499 |
|  | >15 | 1.17 (0.68-1.98) | 0.561 | 1.14 (0.58-2.23) | 0.703 |
| Hypertension grade | Grade 1 & 2 |  |  |  |  |
|  | Grade 3 | 2.10 (1.38-3.27) | **＜0.001***** | 2.09 (1.26-3.57) | **0.005**** |
| Risk classification | Low / Moderate risk |  |  |  |  |
|  | High / Very high risk | 1.70 (0.69-4.78) | 0.277 | 1.04 (0.35-3.39) | 0.950 |
| Coronary heart disease | No |  |  |  |  |
|  | Yes | 0.90 (0.56-1.43) | 0.661 | 0.89 (0.50-1.57) | 0.694 |
| Atherosclerosis | No |  |  |  |  |
|  | Yes | 0.95 (0.70-1.30) | 0.758 | 1.16 (0.79-1.72) | 0.452 |
| Cerebral infarction | No |  |  |  |  |
|  | Yes | 0.96 (0.69-1.33) | 0.799 | 1.09 (0.72-1.63) | 0.685 |
| Coronary muscle bridge | No |  |  |  |  |
|  | Yes | 0.94 (0.65-1.35) | 0.735 | 0.85 (0.56-1.29) | 0.449 |
| Type 2 diabetes mellitus (T2DM) | No |  |  |  |  |
|  | Yes | 1.42 (0.95-2.11) | 0.089 | 0.95 (0.32-2.78) | 0.924 |
| Arrhythmia | No |  |  |  |  |
|  | Yes | 1.06 (0.65-1.71) | 0.808 | 0.94 (0.54-1.63) | 0.826 |
| Alcohol use history | No |  |  |  |  |
|  | Yes | 1.09 (0.77-1.54) | 0.611 | 1.01 (0.63-1.64) | 0.957 |
| Family history | No |  |  |  |  |
|  | Yes | 1.13 (0.83-1.56) | 0.438 | 1.10 (0.76-1.60) | 0.607 |
| Final treatment duration, years | ≤1 |  |  |  |  |
|  | 1–3 | 1.39 (0.88-2.23) | 0.164 | 1.77 (1.06-3.02) | **0.031*** |
|  | 3–5 | 0.95 (0.48-1.87) | 0.888 | 1.27 (0.59-2.72) | 0.534 |
|  | >5 | 0.88 (0.37-1.98) | 0.762 | 1.13 (0.44-2.81) | 0.801 |
| ARB | Not used |  |  |  |  |
|  | Used | 1.39 (0.91-2.18) | 0.136 | 0.98 (0.34-2.85) | 0.974 |
| ACEI | Not used |  |  |  |  |
|  | Used | 1.62 (0.59-4.45) | 0.342 | 1.69 (0.36-7.93) | 0.502 |
| CCB | Not used |  |  |  |  |
|  | Used | 1.73 (1.16-2.61) | **0.009**** | 1.14 (0.41-3.14) | 0.800 |
| Beta-blocker | Not used |  |  |  |  |
|  | Used | 1.03 (0.75-1.40) | 0.872 | 0.73 (0.29-1.80) | 0.491 |
| Alpha-blocker | Not used |  |  |  |  |
|  | Used | 1.53 (0.86-2.71) | 0.141 | 0.98 (0.40-2.35) | 0.964 |
| Diuretic | Not used |  |  |  |  |
|  | Used | 1.51 (0.82-2.76) | 0.179 | 1.09 (0.47-2.52) | 0.842 |
| Combination regimen | Monotherapy |  |  |  |  |
|  | Two drugs | 1.87 (1.16-3.07) | **0.011*** | 1.99 (0.69-5.79) | 0.204 |
|  | Three or more drugs | 2.35 (1.46-3.87) | **＜0.001***** | 2.51 (0.37-17.18) | 0.343 |
| Lipid-lowering agents | Not used |  |  |  |  |
|  | Used | 0.97 (0.65-1.45) | 0.881 | 0.87 (0.53-1.46) | 0.603 |
| Glucose-lowering agents | Not used |  |  |  |  |
|  | Used | 1.39 (0.92-2.09) | 0.120 | 1.44 (0.47-4.40) | 0.518 |
| Passive smoking | No |  |  |  |  |
|  | Yes | 1.37 (0.84-2.24) | 0.206 | 1.38 (0.77-2.47) | 0.278 |
|  | Active smoking | 1.03 (0.73-1.45) | 0.887 | 0.78 (0.47-1.27) | 0.315 |
| Mental stress | Often |  |  |  |  |
|  | Sometimes | 0.73 (0.43-1.22) | 0.224 | 0.69 (0.38-1.23) | 0.210 |
|  | No | 0.52 (0.32-0.84) | **0.008**** | 0.42 (0.22-0.78) | **0.006**** |
| Dietary habit | Salty |  |  |  |  |
|  | Neutral | 0.52 (0.31-0.87) | **0.014*** | 0.43 (0.24-0.79) | **0.006**** |
|  | Light | 0.52 (0.32-0.86) | **0.011*** | 0.50 (0.28-0.91) | **0.022*** |
| Vegetable/fruit intake | Often |  |  |  |  |
|  | Sometimes | 1.34 (0.93-1.94) | 0.116 | 1.23 (0.81-1.88) | 0.334 |
|  | Rarely | 1.44 (0.68-2.99) | 0.331 | 1.29 (0.55-2.98) | 0.560 |
| Exercise frequency (times/week) | No exercise |  |  |  |  |
|  | ≤3 | 0.70 (0.42-1.15) | 0.165 | 0.68 (0.37-1.22) | 0.201 |
|  | >3 | 0.64 (0.45-0.91) | **0.013*** | 0.69 (0.44-1.06) | 0.091 |
| Staying up late | Often |  |  |  |  |
|  | Occasionally | 0.56 (0.32-0.98) | **0.044*** | 0.52 (0.27-0.98) | **0.044*** |
|  | No | 0.58 (0.35-0.96) | **0.032*** | 0.60 (0.31-1.13) | 0.112 |
| Sleep quality | Good |  |  |  |  |
|  | Fair | 0.87 (0.62-1.23) | 0.434 | 0.87 (0.59-1.29) | 0.496 |
|  | Poor | 0.78 (0.48-1.25) | 0.313 | 0.54 (0.31-0.93) | **0.029*** |
| Hcy, μmol/L | – | 1.01 (0.99-1.02) | 0.507 | 0.99 (0.97-1.01) | 0.212 |

Table 5. Multivariable Logistic Regression Analysis of Uncontrolled Blood Pressure with Alternative MTHFR Genotype Grouping (CC vs. CT+TT)

| **Variable** | **Category** | **Univariate logistic regression** | | | **Multivariate logistic regression** | | |
| --- | --- | --- | --- | --- | --- | --- | --- |
|  |  | **OR (95% CI)** | P-value | | **OR (95% CI)** | **P-value** | |
| MTHFR genotype  (CT+TT vs. CC) | CT+TT |  | |  |  | |  |
|  | CC | 0.71 (0.5-1.01) | | 0.058 | 0.58 (0.38-0.88) | | **0.010*** |
| Sex | Male |  | |  |  | |  |
|  | Female | 0.80 (0.56-1.13) | | 0.201 | 0.78 (0.48-1.26) | | 0.303 |
| Ethnicity | Han |  | |  |  | |  |
|  | Uygur | 1.13 (0.79-1.60) | | 0.494 | 1.74 (1.03-2.96) | | **0.038*** |
|  | Other | 1.66 (0.92-2.98) | | 0.089 | 2.34 (1.19-4.63) | | **0.014*** |
| Age, years |  | 0.99 (0.98-1.01) | | 0.243 | 1.00 (0.98-1.03) | | 0.764 |
| BMI, kg/m² |  | 1.04 (0.99-1.08) | | 0.088 | 1.01 (0.96-1.06) | | 0.792 |
| Employment status | Unemployed |  | |  |  | |  |
|  | Employed | 1.19 (0.87-1.64) | | 0.274 | 0.71 (0.39-1.27) | | 0.245 |
| Education level | Junior high school and below |  | |  |  | |  |
|  | Senior high school / Junior college | 1.22 (0.81-1.84) | | 0.350 | 1.12 (0.69-1.82) | | 0.650 |
|  | Bachelor's degree and above | 1.17 (0.76-1.83) | | 0.477 | 0.99 (0.56-1.75) | | 0.959 |
| Work type | Retired / Unemployed |  | |  |  | |  |
|  | Manual worker | 1.26 (0.64-2.43) | | 0.504 | 1.67 (0.72-3.82) | | 0.228 |
|  | Mental worker | 1.37 (0.95-1.98) | | 0.097 | 1.39 (0.83-2.34) | | 0.218 |
|  | Mixed | 0.96 (0.62-1.46) | | 0.836 | NA | | NA |
| Residence | Rural |  | |  |  | |  |
|  | Urban | 0.86 (0.53-1.39) | | 0.522 | 0.79 (0.46-1.37) | | 0.398 |
| Duration of hypertension, years | ≤5 |  | |  |  | |  |
|  | 5–10 | 1.02 (0.69-1.51) | | 0.919 | 0.99 (0.63-1.55) | | 0.970 |
|  | 10–15 | 0.99 (0.57-1.66) | | 0.956 | 0.81 (0.43-1.51) | | 0.512 |
|  | >15 | 1.17 (0.68-1.98) | | 0.561 | 1.13 (0.58-2.21) | | 0.713 |
| Hypertension grade | Grade 1 & 2 |  | |  |  | |  |
|  | Grade 3 | 2.10 (1.38-3.27) | | **＜0.001***** | 2.03 (1.22-3.45) | | **0.008**** |
| Risk classification | Low / Moderate risk |  | |  |  | |  |
|  | High / Very high risk | 1.70 (0.69-4.78) | | 0.277 | 1.12 (0.38-3.66) | | 0.842 |
| Coronary heart disease | No |  | |  |  | |  |
|  | Yes | 0.90 (0.56-1.43) | | 0.661 | 0.86 (0.49-1.52) | | 0.616 |
| Atherosclerosis | No |  | |  |  | |  |
|  | Yes | 0.95 (0.70-1.30) | | 0.758 | 1.14 (0.78-1.69) | | 0.496 |
| Cerebral infarction | No |  | |  |  | |  |
|  | Yes | 0.96 (0.69-1.33) | | 0.799 | 1.10 (0.73-1.65) | | 0.644 |
| Coronary muscle bridge | No |  | |  |  | |  |
|  | Yes | 0.94 (0.65-1.35) | | 0.735 | 0.85 (0.56-1.29) | | 0.460 |
| Type 2 diabetes mellitus | No |  | |  |  | |  |
|  | Yes | 1.42 (0.95-2.11) | | 0.089 | 1.01 (0.34-2.92) | | 0.990 |
| Arrhythmia | No |  | |  |  | |  |
|  | Yes | 1.06 (0.65-1.71) | | 0.808 | 0.93 (0.53-1.60) | | 0.786 |
| Alcohol use history | No |  | |  |  | |  |
|  | Yes | 1.09 (0.77-1.54) | | 0.611 | 0.98 (0.61-1.58) | | 0.936 |
| Family history | No |  | |  |  | |  |
|  | Yes | 1.13 (0.83-1.56) | | 0.438 | 1.10 (0.76-1.60) | | 0.600 |
| Final treatment duration, years | ≤1 |  | |  |  | |  |
|  | 1–3 | 1.39 (0.88-2.23) | | 0.164 | 1.77 (1.06-3.02) | | **0.031*** |
|  | 3–5 | 0.95 (0.48-1.87) | | 0.888 | 1.32 (0.62-2.81) | | 0.472 |
|  | >5 | 0.88 (0.37-1.98) | | 0.762 | 1.16 (0.45-2.89) | | 0.748 |
| ARB | Not used |  | |  |  | |  |
|  | Used | 1.39 (0.91-2.18) | | 0.136 | 1.00 (0.34-2.88) | | 0.995 |
| ACEI | Not used |  | |  |  | |  |
|  | Used | 1.62 (0.59-4.45) | | 0.342 | 1.74 (0.37-8.16) | | 0.479 |
| CCB | Not used |  | |  |  | |  |
|  | Used | 1.73 (1.16-2.61) | | **0.009**** | 1.15 (0.42-3.15) | | 0.787 |
| Beta-blocker | Not used |  | |  |  | |  |
|  | Used | 1.03 (0.75-1.40) | | 0.872 | 0.72 (0.29-1.78) | | 0.477 |
| Alpha-blocker | Not used |  | |  |  | |  |
|  | Used | 1.53 (0.86-2.71) | | 0.141 | 0.97 (0.40-2.32) | | 0.953 |
| Diuretic | Not used |  | |  |  | |  |
|  | Used | 1.51 (0.82-2.76) | | 0.179 | 1.11 (0.48-2.56) | | 0.806 |
| Combination regimen | Monotherapy |  | |  |  | |  |
|  | Two drugs | 1.87 (1.16-3.07) | | **0.011*** | 1.93 (0.69-5.58) | | 0.221 |
|  | Three or more drugs | 2.35 (1.46-3.87) | | **＜0.001***** | 2.48 (0.37-16.75) | | 0.346 |
| Lipid-lowering agents | Not used |  | |  |  | |  |
|  | Used | 0.97 (0.65-1.45) | | 0.881 | 0.89 (0.54-1.47) | | 0.636 |
| Glucose-lowering agents | Not used |  | |  |  | |  |
|  | Used | 1.39 (0.92-2.09) | | 0.120 | 1.35 (0.45-4.09) | | 0.592 |
| Passive smoking | No |  | |  |  | |  |
|  | Yes | 1.37 (0.84-2.24) | | 0.206 | 1.39 (0.78-2.48) | | 0.266 |
|  | Active smoking | 1.03 (0.73-1.45) | | 0.887 | 0.80 (0.49-1.31) | | 0.376 |
| Mental stress | Often |  | |  |  | |  |
|  | Sometimes | 0.73 (0.43-1.22) | | 0.224 | 0.66 (0.37-1.19) | | 0.169 |
|  | No | 0.52 (0.32-0.84) | | **0.008**** | 0.41 (0.22-0.77) | | **0.005**** |
| Dietary habit | Salty |  | |  |  | |  |
|  | Neutral | 0.52 (0.31-0.87) | | **0.014*** | 0.43 (0.24-0.78) | | **0.006**** |
|  | Light | 0.52 (0.32-0.86) | | **0.011*** | 0.49 (0.27-0.89) | | **0.019*** |
| Vegetable/fruit intake | Often |  | |  |  | |  |
|  | Sometimes | 1.34 (0.93-1.94) | | 0.116 | 1.19 (0.78-1.81) | | 0.408 |
|  | Rarely | 1.44 (0.68-2.99) | | 0.331 | 1.22 (0.52-2.83) | | 0.643 |
| Exercise frequency (times/week) | No exercise |  | |  |  | |  |
|  | ≤3 | 0.70 (0.42-1.15) | | 0.165 | 0.69 (0.38-1.24) | | 0.221 |
|  | >3 | 0.64 (0.45-0.91) | | **0.013*** | 0.70 (0.45-1.08) | | 0.111 |
| Staying up late | Often |  | |  |  | |  |
|  | Occasionally | 0.56 (0.32-0.98) | | **0.044*** | 0.53 (0.28-1.00) | | **0.050*** |
|  | No | 0.58 (0.35-0.96) | | **0.032*** | 0.60 (0.32-1.13) | | 0.112 |
| Sleep quality | Good |  | |  |  | |  |
|  | Fair | 0.87 (0.62-1.23) | | 0.434 | 0.90 (0.61-1.31) | | 0.574 |
|  | Poor | 0.78 (0.48-1.25) | | 0.313 | 0.55 (0.31-0.94) | | **0.032*** |
| Hcy, μmol/L |  | 1.01 (0.99-1.02) | | 0.507 | 1.00 (0.98-1.01) | | 0.615 |

Table 6. Univariate and multivariable logistic regression analysis of LASSO-selected predictors associated with uncontrolled blood pressure

| **Variable** | **Category** | **Univariate logistic regression** | | | **Multivariate logistic regression** | | |
| --- | --- | --- | --- | --- | --- | --- | --- |
|  |  | **OR (95% CI)** | | P-value | **OR (95% CI)** | | **P-value** |
| MTHFR genotype | CC |  |  | |  |  | |
|  | CT | 1.26 (0.86–1.85) | 0.236 | | 1.43 (0.95–2.17) | 0.089 | |
|  | TT | 1.74 (1.13–2.70) | **0.012*** | | 2.06 (1.28–3.34) | **0.003**** | |
| Ethnicity | Han |  |  | |  |  | |
|  | Uygur | 1.13 (0.79–1.60) | 0.494 | | 1.49 (0.94–2.35) | 0.091 | |
|  | Other | 1.66 (0.92–2.98) | 0.089 | | 2.05 (1.07–3.94) | **0.029*** | |
| BMI, kg/m² | – | 1.04 (0.99–1.08) | 0.088 | | 1.01 (0.97–1.06) | 0.566 | |
| Hypertension grade | Grade 1 & 2 |  |  | |  |  | |
|  | Grade 3 | 2.10 (1.38–3.27) | **＜**0.001*** | | 1.93 (1.22–3.11) | **0.006**** | |
| T2DM | No |  |  | |  |  | |
|  | Yes | 1.42 (0.95–2.11) | 0.089 | | 0.95 (0.33–2.67) | 0.924 | |
| CCB | No |  |  | |  |  | |
|  | Yes | 1.73 (1.16–2.61) | **0.009**** | | 1.21 (0.68–2.16) | 0.507 | |
| Beta-blocker | No |  |  | |  |  | |
|  | Yes | 1.03 (0.75–1.40) | 0.872 | | 0.73 (0.44–1.19) | 0.207 | |
| Antihypertensive combination regimen | Monotherapy |  |  | |  |  | |
|  | Two drugs | 1.87 (1.16–3.07) | **0.011*** | | 1.79 (1.01–3.23) | 0.051 | |
|  | Three or more drugs | 2.35 (1.46–3.87) | **＜**0.001*** | | 2.24 (1.01–5.10) | **0.049*** | |
| Glucose-lowering drugs | No |  |  | |  |  | |
|  | Yes | 1.39 (0.92–2.09) | 0.120 | | 1.37 (0.47–4.01) | 0.559 | |
| Psychological stress | Often |  |  | |  |  | |
|  | Sometimes | 0.73 (0.43–1.22) | 0.224 | | 0.66 (0.38–1.15) | 0.144 | |
|  | No | 0.52 (0.32–0.84) | **0.008**** | | 0.45 (0.26–0.78) | **0.004**** | |
| Dietary pattern | Salty |  |  | |  |  | |
|  | Neutral | 0.52 (0.31–0.87) | **0.014*** | | 0.47 (0.26–0.83) | **0.010**** | |
|  | Light | 0.52 (0.32–0.86) | **0.011*** | | 0.53 (0.31–0.93) | **0.026*** | |
| Vegetable and fruit intake | Often |  |  | |  |  | |
|  | Sometimes | 1.34 (0.93–1.94) | 0.116 | | 1.16 (0.78–1.73) | 0.466 | |
|  | Rarely | 1.44 (0.68–2.99) | 0.331 | | 1.33 (0.60–2.91) | 0.478 | |
| Exercise frequency (times/week) | No exercise |  |  | |  |  | |
|  | ≤3 times/week | 0.70 (0.42–1.15) | 0.165 | | 0.70 (0.40–1.22) | 0.212 | |
|  | >3 times/week | 0.64 (0.45–0.91) | **0.013*** | | 0.68 (0.45–1.02) | 0.063 | |
| Sleep quality | Good |  |  | |  |  | |
|  | Fair | 0.87 (0.62–1.23) | 0.434 | | 0.88 (0.61–1.27) | 0.487 | |
|  | Poor | 0.78 (0.48–1.25) | 0.313 | | 0.60 (0.35–1.00) | 0.053 | |

Table 7. Univariate and Multivariable Logistic Regression Analysis of LASSO-Selected Predictors Associated with Uncontrolled Blood Pressure (CC vs CT+TT)

| **Variable** | **Category** | **Univariate logistic regression** | | **Multivariate logistic regression** | |
| --- | --- | --- | --- | --- | --- |
|  |  | **OR (95% CI)** | **P-value** | **OR (95% CI)** | **P-value** |
| MTHFR genotype  (CT+TT vs. CC) | CC |  |  |  |  |
|  | CT+TT | 1.41 (0.99–2.02) | 0.058 | 1.64 (1.11–2.45) | **0.014*** |
| Ethnicity | Han |  |  |  |  |
|  | Uygur | 1.13 (0.79–1.60) | 0.494 | 1.46 (0.93–2.32) | 0.104 |
|  | Other | 1.66 (0.92–2.98) | 0.089 | 2.14 (1.12–4.09) | 0.020* |
| BMI, kg/m² |  | 1.04 (0.99–1.08) | 0.088 | 1.01 (0.97–1.06) | 0.671 |
| Hypertension grade | Grade 1 & 2 |  |  |  |  |
|  | Grade 3 | 2.10 (1.38–3.27) | **<0.001***** | 1.91 (1.21–3.07) | **0.007**** |
| T2DM | No |  |  |  |  |
|  | Yes | 1.42 (0.95–2.11) | 0.089 | 1.03 (0.37–2.87) | 0.951 |
| CCB | Not used |  |  |  |  |
|  | Used | 1.73 (1.16–2.61) | **0.009**** | 1.19 (0.67–2.12) | 0.549 |
| β-blocker | Not used |  |  |  |  |
|  | Used | 1.03 (0.75–1.40) | 0.872 | 0.70 (0.43–1.15) | 0.158 |
| Antihypertensive combination | Monotherapy |  |  |  |  |
|  | Two drugs | 1.87 (1.16–3.07) | **0.011*** | 1.82 (1.02–3.29) | **0.045*** |
|  | Three or more drugs | 2.35 (1.46–3.87) | **<0.001***** | 2.38 (1.07–5.43) | **0.035*** |
| Glucose-lowering drugs | Not used |  |  |  |  |
|  | Used | 1.39 (0.92–2.09) | 0.120 | 1.27 (0.44–3.69) | 0.653 |
| Mental stress | Often |  |  |  |  |
|  | Sometimes | 0.73 (0.43–1.22) | 0.224 | 0.65 (0.38–1.14) | 0.133 |
|  | None | 0.52 (0.32–0.84) | **0.008**** | 0.44 (0.25–0.77) | **0.004**** |
| Dietary pattern | Salty |  |  |  |  |
|  | Neutral | 0.52 (0.31–0.87) | **0.014*** | 0.45 (0.25–0.81) | **0.007**** |
|  | Light | 0.52 (0.32–0.86) | **0.011*** | 0.51 (0.29–0.89) | **0.018*** |
| Vegetable/fruit intake | Often |  |  |  |  |
|  | Sometimes | 1.34 (0.93–1.94) | 0.116 | 1.12 (0.75–1.68) | 0.565 |
|  | Rarely | 1.44 (0.68–2.99) | 0.331 | 1.27 (0.57–2.78) | 0.549 |
| Exercise frequency, times/week | No exercise |  |  |  |  |
|  | ≤3 | 0.70 (0.42–1.15) | 0.165 | 0.70 (0.39–1.21) | 0.202 |
|  | >3 | 0.64 (0.45–0.91) | **0.013*** | 0.68 (0.45–1.03) | 0.067 |
| Sleep quality | Good |  |  |  |  |
|  | Fair | 0.87 (0.62–1.23) | 0.434 | 0.88 (0.61–1.27) | 0.492 |
|  | Poor | 0.78 (0.48–1.25) | 0.313 | 0.59 (0.35–0.99) | **0.048*** |
| Hcy, μmol/L |  | 1.01 (0.99–1.02) | 0.507 | 1.00 (0.98–1.01) | 0.580 |

Table 8. Conditional Logistic Regression Analysis of Uncontrolled Blood Pressure with MTHFR Genotype Grouping (CC vs. CT+TT) in the Propensity Score-Matched Cohort

| **Variable** | **Category** | **Univariate logistic regression** | | | **Multivariate logistic regression** | | |
| --- | --- | --- | --- | --- | --- | --- | --- |
|  |  | **OR (95% CI)** | P-value | | **OR (95% CI)** | **P-value** | |
| MTHFR genotype  (CC vs. CT+TT) | CT+TT |  | |  |  | |  |
|  | CC | 0.62 (0.42-0.92) | | **0.017*** | 0.63 (0.41-0.96) | | **0.031*** |
| Ethnicity | Han |  | |  |  | |  |
|  | Uygur | 1.46 (0.92-2.32) | | 0.105 | 1.44 (0.86-2.43) | | 0.168 |
|  | Other | 2.03 (1.06-3.90) | | **0.032*** | 1.92 (0.89-4.13) | | 0.093 |
| BMI classification | <24 |  | |  |  | |  |
|  | 24–28 | 0.67 (0.42-1.07) | | 0.094 | 0.62 (0.36-1.09) | | 0.093 |
|  | ≥28 | 0.89 (0.55-1.45) | | 0.648 | 0.71 (0.40-1.26) | | 0.239 |
| Hypertension grade | Grade 1 & 2 |  | |  |  | |  |
|  | Grade 3 | 1.98 (1.25-3.20) | | **0.004**** | 2.46 (1.40-4.53) | | **0.003**** |
| T2DM | Yes |  | |  |  | |  |
|  | No | 1.02 (0.36-2.82) | | 0.977 | 0.66 (0.16-2.48) | | 0.550 |
| CCB | Not used |  | |  |  | |  |
|  | Used | 1.18 (0.66-2.09) | | 0.578 | 1.27 (0.65-2.49) | | 0.478 |
| Beta-blocker | Used |  | |  |  | |  |
|  | Not used | 0.69 (0.42-1.12) | | 0.133 | 0.68 (0.38-1.21) | | 0.192 |
| Antihypertensive combination regimen | Monotherapy |  | |  |  | |  |
|  | Two drugs | 1.79 (1.01-3.24) | | **0.050*** | 1.64 (0.85-3.23) | | 0.146 |
|  | Three or more drugs | 2.40 (1.08-5.45) | | **0.034*** | 1.98 (0.79-5.07) | | 0.150 |
| Glucose-lowering drugs | Used |  | |  |  | |  |
|  | Not used | 1.29 (0.45-3.74) | | 0.637 | 2.35 (0.62-9.79) | | 0.218 |
| Psychological stress | Often |  | |  |  | |  |
|  | Sometimes | 0.64 (0.37-1.12) | | 0.122 | 0.70 (0.34-1.44) | | 0.329 |
|  | Rarely | 0.44 (0.25-0.76) | | **0.004**** | 0.50 (0.25-1.02) | | 0.056 |
| Dietary pattern | Salty |  | |  |  | |  |
|  | Neutral | 0.46 (0.26-0.82) | | **0.009**** | 0.36 (0.18-0.73) | | **0.005**** |
|  | Light | 0.51 (0.29-0.89) | | **0.019*** | 0.42 (0.21-0.82) | | **0.012*** |
| Vegetable and fruit intake | Often |  | |  |  | |  |
|  | Sometimes | 1.12 (0.75-1.67) | | 0.568 | 1.24 (0.77-1.97) | | 0.374 |
|  | Rarely | 1.22 (0.55-2.67) | | 0.620 | 1.52 (0.54-4.23) | | 0.415 |
| Exercise frequency (times/week) | No exercise |  | |  |  | |  |
|  | <3 times/week | 0.72 (0.41-1.25) | | 0.247 | 0.65 (0.31-1.31) | | 0.236 |
|  | >3 times/week | 0.70 (0.46-1.06) | | 0.094 | 0.58 (0.34-0.95) | | **0.033*** |
| Sleep quality | Good |  | |  |  | |  |
|  | Fair | 0.87 (0.60-1.26) | | 0.460 | 0.96 (0.62-1.48) | | 0.852 |
|  | Poor | 0.58 (0.34-0.98) | | **0.043*** | 0.45 (0.23-0.84) | | **0.014*** |

Table 9. IPW-Weighted Multivariable Logistic Regression Analysis of Uncontrolled Blood Pressure Using the Additive Genetic Model (MTHFR C677T: CC vs CT vs TT)

| **Variable** | **Category** | **Univariate logistic regression** | | | **Multivariate logistic regression** | | |
| --- | --- | --- | --- | --- | --- | --- | --- |
|  |  | **OR (95% CI)** | P-value | | **OR (95% CI)** | **P-value** | |
| MTHFR C677T | CC |  | |  |  | |  |
|  | CT | 1.26 (0.86–1.85) | | 0.236 | 1.43 (1.11–1.83) | | **0.005**** |
|  | TT | 1.74 (1.13–2.70) | | **0.012*** | 2.09 (1.62–2.70) | | **<0.001***** |
| Ethnicity | Han |  | |  |  | |  |
|  | Uygur | 1.13 (0.79–1.60) | | 0.494 | 1.00 (0.75–1.32) | | 0.973 |
|  | Other | 1.66 (0.92–2.98) | | 0.089 | 2.39 (1.61–3.56) | | **<0.001***** |
| BMI, kg/m² | – | 1.04 (0.99–1.08) | | 0.088 | 1.03 (1.01–1.06) | | **0.011*** |
| Hypertension grade | Grade 1 & 2 |  | |  |  | |  |
|  | Grade 3 | 2.10 (1.38–3.27) | | **<0.001***** | 2.03 (1.51–2.75) | | **<0.001***** |
| T2DM | No |  | |  |  | |  |
|  | Yes | 1.42 (0.95–2.11) | | 0.089 | 1.42 (0.66–3.09) | | 0.371 |
| CCB | Not used |  | |  |  | |  |
|  | Used | 1.73 (1.16–2.61) | | **0.009**** | 1.31 (0.89–1.92) | | 0.174 |
| β-blocker | Not used |  | |  |  | |  |
|  | Used | 1.03 (0.75–1.40) | | 0.872 | 0.60 (0.44–0.82) | | 0**.001**** |
| Antihypertensive combination | Monotherapy |  | |  |  | |  |
|  | Two drugs | 1.87 (1.16–3.07) | | **0.011*** | 2.37 (1.61–3.52) | | **<0.001***** |
|  | Three or more drugs | 2.35 (1.46–3.87) | | **<0.001***** | 3.24 (1.91–5.58) | | **<0.001***** |
| Glucose-lowering drugs | Not used |  | |  |  | |  |
|  | Used | 1.39 (0.92–2.09) | | 0.12 | 1.23 (0.56–2.69) | | 0.61 |
| Mental stress | Often |  | |  |  | |  |
|  | Sometimes | 0.73 (0.43–1.22) | | 0.224 | 0.76 (0.55–1.05) | | 0.094 |
|  | None | 0.52 (0.32–0.84) | | **0.008**** | 0.53 (0.39–0.74) | | **<0.001***** |
| Dietary pattern | Salty |  | |  |  | |  |
|  | Neutral | 0.52 (0.31–0.87) | | **0.014*** | 0.44 (0.30–0.64) | | **<0.001***** |
|  | Light | 0.52 (0.32–0.86) | | **0.011*** | 0.50 (0.35–0.71) | | **<0.001***** |
| Vegetable/fruit intake | Often |  | |  |  | |  |
|  | Sometimes | 1.34 (0.93–1.94) | | 0.116 | 0.85 (0.66–1.10) | | 0.215 |
|  | Rarely | 1.44 (0.68–2.99) | | 0.331 | 0.96 (0.58–1.58) | | 0.867 |
| Exercise frequency, times/week | No exercise |  | |  |  | |  |
|  | ≤3 | 0.70 (0.42–1.15) | | 0.165 | 0.63 (0.44–0.89) | | **0.009**** |
|  | >3 | 0.64 (0.45–0.91) | | **0.013*** | 0.60 (0.47–0.77) | | **<0.001***** |
| Sleep quality | Good |  | |  |  | |  |
|  | Fair | 0.87 (0.62–1.23) | | 0.434 | 0.68 (0.54–0.85) | | **<0.001***** |
|  | Poor | 0.78 (0.48–1.25) | | 0.313 | 0.48 (0.35–0.66) | | **<0.001***** |

Table 10. Basic characteristics of studies included in the meta-analysis

| **Study** | **Year** | **Age**  **(years)** | **Sex**  **(male/female)** | **Follow-up**  **(months)** | **Intervention** | **Outcomes** | **CC**  **(n)** | **CT**  **(n)** | **TT**  **(n)** |
| --- | --- | --- | --- | --- | --- | --- | --- | --- | --- |
| Wu HH et al. | 2023 | NR | NR | 1, 3, 6, 12 | Enalapril maleate/folic acid (10 mg/0.8 mg) | ΔHcy, ΔSBP | 100 | 193 | 139 |
| Liang XW et al. | 2022 | CC: 59.2±13.5 | CC: 26/29 | 3 | Enalapril maleate/folic acid (10 mg/0.8 mg) | ΔHcy | 55 | 96 | 52 |
|  |  | CT: 59.0±14.0 | CT: 62/34 |  |  |  |  |  |  |
|  |  | TT: 53.2±12.6 | TT: 26/26 |  |  |  |  |  |  |
| Chen P et al. | 2022 | CC: 61.54±6.82 | CC: 29/21 | 1, 3 | Conventional antihypertensive drugs + folic acid 5 mg | ΔHcy | 50 | 74 | 54 |
|  |  | CT: 60.45±7.42 | CT: 42/32 |  |  |  |  |  |  |
|  |  | TT: 58.66±8.23 | TT: 31/23 |  |  |  |  |  |  |
| Jia XY | 2021 | 58.1±8.4 | 60/53 | 3 | Enalapril maleate/folic acid (10 mg/0.8 mg) | ΔHcy, ΔSBP | 28 | 48 | 37 |
| Liu ZQ et al. | 2021 | NR | NR | 6 | Conventional antihypertensive drugs + folic acid 0.8 mg | ΔHcy, ΔSBP | 31 | 30 | 60 |
| Zhang YF et al. | 2020 | CC: 38.5±7.6 | CC: 31/22 | 6 | Conventional antihypertensive drugs + folic acid 5 mg | ΔHcy | 53 | 119 | 88 |
|  |  | CT: 37.1±6.8 | CT: 63/56 |  |  |  |  |  |  |
|  |  | TT: 39.3±8.4 | TT: 49/39 |  |  |  |  |  |  |
| Qiu DX | 2019 | CC: 57.96±10.37 | CC: 13/11 | 3 | Conventional antihypertensive drugs + folic acid 0.8 mg | ΔHcy, ΔSBP | 24 | 28 | 50 |
|  |  | CT: 54.97±12.06 | CT: 20/8 |  |  |  |  |  |  |
|  |  | TT: 56.07±11.14 | TT: 31/19 |  |  |  |  |  |  |
| Chen GK et al. | 2019 | 58.19±2.24 | 103/77 | 1 | Enalapril maleate/folic acid (5 mg/0.4 mg) | ΔHcy | 59 | 56 | 65 |
| Shen TT et al. | 2019 | 80.9±11.2 | 37/23 | 1 | Valsartan + folic acid 5 mg | ΔHcy | 17 | 16 | 27 |
| Liu P et al. | 2018 | NR | NR | 3 | Folic acid 0.8 mg | ΔHcy | 169 | 92 | 89 |
| Zhang C et al. | 2018 | 74.5±13.1 | 87/59 | 4 | Conventional antihypertensive drugs + folic acid 5 mg + vitamin B12 5 mg | ΔHcy | 26 | 46 | 74 |
| Dong L et al. | 2017 | 75.6±10.2 | 73/49 | 3 | Conventional antihypertensive drugs + folic acid 5 mg | ΔHcy, ΔSBP | 26 | 51 | 45 |
| Xia HY et al. | 2017 | 61.7±15.6 | 44/41 | 1 | Enalapril maleate/folic acid (10 mg/0.8 mg) | ΔHcy | 32 | 29 | 24 |
| Xie S | 2016 | 58.8±19.7 | 121/111 | 1, 2, 3 | Enalapril maleate/folic acid (10 mg/0.8 mg) | ΔHcy | 69 | 93 | 70 |
| Wang TT et al. | 2014 | NR | NR | 3 | Conventional antihypertensive drugs + folic acid 0.8 mg | ΔHcy, ΔSBP | 42 | 42 | 82 |

**Relationship between TT type and CC type Hcy changes of MTHFR C677T**

Table 11. Mediator Model — TT Genotype → Plasma Hcy (Linear Regression)

| Variable | β | SE | t | P |
| --- | --- | --- | --- | --- |
| TT genotype (vs. CC+CT) | 9.591 | 0.814 | 11.79 | **<0.001***** |
| Age (years) | −0.035 | 0.034 | −1.05 | 0.295 |
| Sex (female vs. male) | −3.189 | 0.802 | −3.98 | **<0.001***** |
| BMI 24–28 (vs. <24) | −1.715 | 0.96 | −1.79 | 0.075 |
| BMI ≥28 (vs. <24) | −1.600 | 1.003 | −1.60 | 0.111 |
| Grade 3 HTN (vs. Grade 1 & 2) | 0.492 | 0.877 | 0.56 | 0.576 |
| Stress: sometimes (vs. often) | 1.375 | 1.174 | 1.17 | 0.242 |
| Stress: none (vs. often) | 0.934 | 1.167 | 0.8 | 0.424 |
| Diet: neutral (vs. salty) | −2.748 | 1.183 | −2.32 | **0.021*** |
| Diet: light (vs. salty) | −2.416 | 1.148 | −2.10 | **0.036*** |
| Exercise: <3/wk (vs. none) | −1.787 | 1.1 | −1.62 | 0.105 |
| Exercise: ≥3/wk (vs. none) | −1.216 | 0.779 | −1.56 | 0.119 |

Table 12. Outcome Model — Association of TT Genotype and Hcy with Uncontrolled Blood Pressure (Logistic Regression)

| Variable | β | SE | OR | 95% CI | P |
| --- | --- | --- | --- | --- | --- |
| TT genotype (vs. CC+CT) | 0.548 | 0.214 | 1.73 | 1.14–2.63 | **0.011*** |
| Hcy (per 1 μmol/L) | −0.010 | 0.01 | 0.99 | 0.97–1.01 | 0.276 |
| Age (years) | 0.003 | 0.008 | 1 | 0.99–1.02 | 0.711 |
| Sex (female vs. male) | −0.168 | 0.198 | 0.85 | 0.57–1.25 | 0.397 |
| BMI 24–28 (vs. <24) | −0.328 | 0.234 | 0.72 | 0.45–1.14 | 0.161 |
| BMI ≥28 (vs. <24) | 0.099 | 0.24 | 1.1 | 0.69–1.76 | 0.680 |
| Grade 3 HTN (vs. Grade 1 & 2) | 0.798 | 0.23 | 2.22 | 1.41–3.48 | **<0.001***** |
| Stress: sometimes (vs. often) | −0.301 | 0.276 | 0.74 | 0.43–1.27 | 0.275 |
| Stress: none (vs. often) | −0.655 | 0.277 | 0.52 | 0.30–0.89 | **0.018*** |
| Diet: neutral (vs. salty) | −0.697 | 0.282 | 0.5 | 0.29–0.87 | **0.013*** |
| Diet: light (vs. salty) | −0.626 | 0.272 | 0.53 | 0.31–0.91 | **0.022*** |
| Exercise: <3/wk (vs. none) | −0.478 | 0.27 | 0.62 | 0.37–1.05 | 0.077 |
| Exercise: ≥3/wk (vs. none) | −0.396 | 0.19 | 0.67 | 0.46–0.98 | **0.037*** |
